# Gametocyte carriage in an era of changing malaria epidemiology: A 19-year analysis of a malaria longitudinal cohort

**DOI:** 10.12688/wellcomeopenres.15186.2

**Published:** 2019-05-28

**Authors:** Michelle K. Muthui, Polycarp Mogeni, Kennedy Mwai, Christopher Nyundo, Alex Macharia, Thomas N. Williams, George Nyangweso, Juliana Wambua, Daniel Mwanga, Kevin Marsh, Philip Bejon, Melissa C. Kapulu

**Affiliations:** 1 Department of Biosciences, KEMRI-Wellcome Trust Research Programme, Kilifi, 230-80108, Kenya; 2 African Health Research Institute, Durban, Congella, 4013, Private bag X7, South Africa; 3 Epidemiology and Biostatistics Division, School of Public Health, University of the Witwatersrand, Johannesburg, Parktown, 2193, 27 St Andrews Road, South Africa; 4 Department of Medicine, Imperial College London, St Mary's Campus, London, W21NY, UK; 5 Centre for Tropical Medicine and Global Health, Nuffield Department of Clinical Medicine, University of Oxford, Oxford, OX3 7FZ, UK

**Keywords:** malaria, Plasmodium falciparum, gametocyte carriage, artemisinin combination therapy

## Abstract

**Background: **Interventions to block malaria transmission from humans to mosquitoes are currently in development. To be successfully implemented, key populations need to be identified where the use of these transmission-blocking and/or reducing strategies will have greatest impact.

**Methods: **We used data from a longitudinally monitored cohort of children from Kilifi county located along the Kenyan coast collected between 1998-2016 to describe the distribution and prevalence of gametocytaemia in relation to transmission intensity, time and age. Data from 2,223 children accounting for 9,134 person-years of follow-up assessed during cross-sectional surveys for asexual parasites and gametocytes were used in logistic regression models to identify factors predictive of gametocyte carriage in this cohort.

**Results: **Our analysis showed that children 1-5 years of age were more likely to carry microscopically detectable gametocytes than their older counterparts. Carrying asexual parasites and recent episodes of clinical malaria were also strong predictors of gametocyte carriage. The prevalence of asexual parasites and of gametocyte carriage declined over time, and after 2006, when artemisinin combination therapy (ACT) was introduced, recent episodes of clinical malaria ceased to be a predictor of gametocyte carriage.

**Conclusions: **Gametocyte carriage in children in Kilifi has fallen over time.  Previous episodes of clinical malaria may contribute to the development of carriage, but this appears to be mitigated by the use of ACTs highlighting the impact that gametocidal antimalarials can have in reducing the overall prevalence of gametocytaemia when targeted on acute febrile illness.

## Introduction

Considerable progress has been made towards eliminating malaria over the years, with an unprecedented reduction in disease burden between 2000 to 2010, albeit with progress stalling between 2010 to 2015 ^1^. Causality is complex, but reductions have been attributed to the increased use of insecticide treated nets and the adoption of highly effective artemisinin combination therapies (ACTs) as the first line treatment for malaria ^2– 4^.

Malaria is transmitted via gametocytes taken up during a blood meal by female *Anopheles* mosquitoes. Gametocytes are produced when a proportion of the asexual parasites, an average of 1 gametocyte per 156 asexual parasites ^5^, commit to sexual development during a malaria infection. It is not yet clear what factors drive this commitment but it has been proposed that factors such as drug pressure, an unfavourable environment within the host including host immunity ^6, 7^ and parasite factors such as the contents of extracellular vesicles released from infected erythrocytes ^8, 9^ may play a role. Genetic influences, in particular, variants of the β-globin locus have also been shown to influence gametocyte production in asymptomatic infections where variants that protect against severe malaria ^10^ are associated with an increased rate of gametocyte production ^11, 12^.

Older antimalarials such as chloroquine (CQ) and sulfadoxine-pyrimethamine (SP) were active primarily against asexual parasites and had limited activity against gametocytes, particularly when resistance emerged ^13, 14^. Primaquine is active against mature gametocytes, and the World Health Organisation (WHO) recommends a single low dose of 0.25 mg/kg primaquine for use in low transmission areas to reduce malaria transmission ^15^. Although not active against the mature gametocytes, ACTs act against early stage gametocytes and consequently reduce gametocyte carriage ^4, 6^.

Identifying prognostic indicators of gametocyte carriage is key to the successful implementation of interventions aimed at reducing malaria transmission. Several studies have examined the epidemiology of gametocyte carriage but these have largely been single surveys ^14, 16– 18^ or limited to short-term follow up ^19, 20^. We carried out an analysis of data collected over 19 years of follow-up in a longitudinal cohort established at the Kenyan coast, during a period of changing malaria transmission and changing drug use. Here, we sought to describe the distribution and prevalence of *Plasmodium falciparum* gametocyte carriage within this cohort and changing prevalence over time, and thus identify potential risk factors for gametocyte carriage.

## Methods

### Study design and data collection

**Figure 1.**
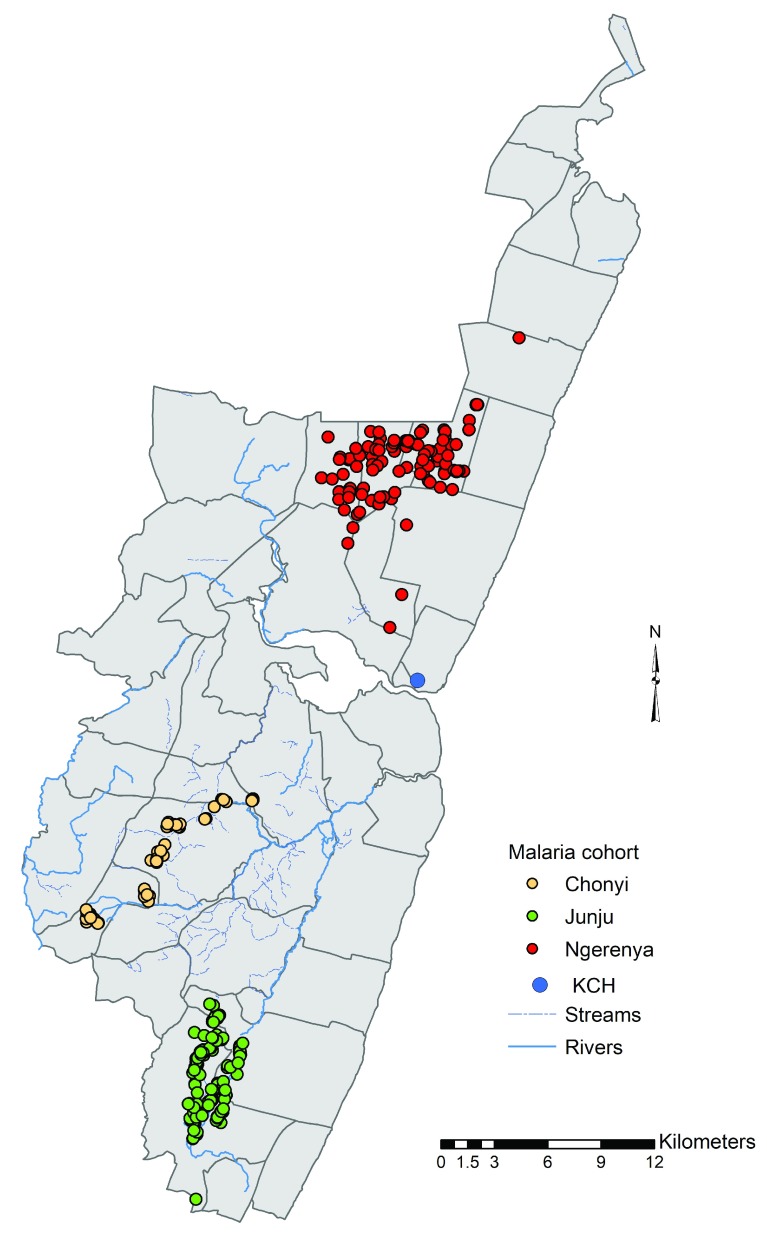
Map of the Kilifi Malaria Longitudinal Cohort study area located within the Kilifi Health and Demographic Surveillance System (KHDSS). Coloured points show the location of the participants homesteads within the three cohorts, Chonyi (orange); Junju (green); and Ngerenya (red). KCH, Kilifi County Hospital.

Cohorts of children recruited into the Kilifi Malaria Longitudinal Cohort study were located in Kilifi County at the Kenyan coast ( Figure 1) ^21– 23^. Three cohorts located in areas of varying transmission intensity were included, that is, Ngerenya (initially moderate transmission but falling to low transmission), Junju (moderate transmission) and Chonyi (high transmission). Malaria transmission intensity is higher during the rainy seasons with the long rainy season occurring between May-July and short rainy season between October-December ^21, 24^. We analysed data from cross-sectional surveys conducted within the three cohorts. Data included in the analyses were from cross-sectional surveys conducted from 1998 to 2016 for Ngerenya (a cross-sectional survey was not conducted in 2006); from 1999 to 2001 for Chonyi; and from 2007 to 2016 for Junju.

In Ngerenya and Chonyi, households were selected randomly with 72 households selected in Ngerenya (819 participants) and 52 households in Chonyi (783) participants ^21, 22^. This sample size was considered appropriate for a study on the definitions of clinical malaria. Participants willing to continue follow-up were included as a pragmatic study size for future studies. For Junju, participants were recruited from 405 children who previously participated in a malaria vaccine trial ^23^. The sample size was determined based on an expected febrile malaria incidence of 50%. Enrolling 400 children would then allow detection of 35% vaccine efficacy with 80% power. Children born into the households were then subsequently recruited into the three cohorts over time. All study participants had access to healthcare facilities with the same study protocol applied to all cohorts per year. Children were actively monitored for malaria by weekly visits to identify febrile episodes and by cross-sectional surveys for asymptomatic parasitaemia, and exited follow up when they were 15 years of age ^25^.

To reflect the marked decline in transmission intensity observed in Ngerenya over the follow-up period, for analytical purposes, Ngerenya was divided into Ngerenya early, which included data collected during a period of moderate transmission (1998–2001), and Ngerenya late, which included data collected during the period of moderate to low transmission (2002–2016) ^26^.

### Ethics approval and consent to participate

Approval for participation in these cohort studies was given by Kenya Medical Research Institute Ethics Review Committee (reference numbers KEMRI/SERU/CGMRC//3149 and SSC1131), and research was conducted according to the principles of the Declaration of Helsinki, which included the administration of informed consenting in the participant’s local language prior to any study procedure. Written informed consent for participation in this study was provided by the parents of the children included in this study.

### Case detection

Active malaria surveillance was performed during weekly follow-up visits, carried out as described previously ^21, 27^. Briefly, households in the three cohorts were visited by a field worker every week where axillary temperature was recorded for each study participant. If the participant had a fever or history of fever, they had a blood smear performed by the field worker to diagnose malaria infection. From 2007 onwards, rapid diagnostic tests (RDTs) were available in the field to guide treatment decisions, but even before RDTs were available all febrile malaria episodes were treated. Field workers were resident in the villages where study participants lived, and were available to assess febrile episodes arising before a scheduled visit was planned. Treatment was freely provided by the field workers and the drug administered in a particular year was based on the Government of Kenya national guidelines for treatment of malaria.

### Cross-sectional parasitological surveys

To analyse gametocyte and asexual parasite prevalence, data from cross-sectional surveys mainly taken before the beginning of the long rainy season to assess asymptomatic *P. falciparum* infections were used. There were approximately 364 (range, 139–556) participants in each survey, and a detailed summary of the cross-sectional surveys included in the analysis with the number of participants attending each survey is presented in Supplementary Table 1 ^28^.

### Laboratory investigations

Thick and thin blood films were taken from all children at each cross-sectional survey and for children presenting with fever during the weekly follow-up visits. The thin blood films were fixed with 100% methanol and stained with 3% Giemsa stain for 45 minutes before being examined for parasites. Thick films were air-dried before staining. If there were more than 25 parasites per high powered field on the thick film then the thin film was used for counting, otherwise the thick film was used. Asexual parasite densities were determined per microliter of blood and were calculated as the number of parasites per 200 white blood cells (WBCs) for thick films or per 500 red blood cells (RBCs) for thin films. The final parasitaemia was then calculated in reference to the actual full blood count (if available) or estimated assuming a WBC count of 8 × 10 ^9^ per litre or an RBC count of 5 × 10 ^12^ per litre. In total, 100 high-powered fields of a thick film were read before ascertaining that no parasites were present.

Gametocytes were counted when observed during application of the protocol for asexual parasites, and hence the numbers of fields examined during which gametocytes may be observed varied depending on the asexual parasitaemia. Malaria parasite and gametocyte counts were determined by two independent readers and discordant readings resolved by a third reader. Quality assurance over the study period included comprehensive microscopy training during induction and at regular intervals using internal and external quality control. For internal quality control, a subset of slides selected quarterly are re-read by the microscopy team and concordance between the results checked. For external quality control, at the beginning of the cohort study, this involved reading reference blood films from a partner lab in the United Kingdom. Currently, external quality control involves participating in three annual surveys carried out by the National Institute of Communicable Diseases (NICD) based in South Africa where they send 20 slides per survey to our lab for proficiency testing.

A subset of samples were typed for sickle cell genotype and α-thalassaemia, as previously described ^29, 30^.

### Case definitions

To determine malaria episodes, data from the weekly follow-up visits were used and malaria episodes defined using previously described cut-offs ^21^. For the weekly follow-up visits, temperature was recorded for the participants, and for those with a fever, a blood film was taken and analysed as above. For children <1 year of age, clinical malaria was defined as a fever (axillary temperature ≥37.5°C) with any parasitaemia while for children between 1–15 years of age malaria was defined as fever accompanied by parasitaemia of ≥2,500 parasites/µl of blood. For estimates of the number of malaria episodes per participant per survey, malaria episodes were considered as unique only if the time difference between two consecutive malaria episodes was ≥28 days. Malaria episodes occurring in the interval between two cross-sectional surveys, the respective survey (survey x) and the prior survey (survey x-1), were identified, summed up and defined as the number of malaria episodes occurring in the period leading up to each respective survey.

### Statistical analysis

To assess the relationship between variables, Spearman’s rank correlation coefficients were calculated. Models to predict gametocyte positivity were fitted using the following known covariates shown to be associated with gametocyte carriage ^6, 31^: asexual parasite positivity, age, year, number of malaria episodes and whether the participant had a malaria episode, asexual parasite positive blood film or gametocyte positive blood film in the prior cross-sectional survey. The variable asexual parasite positive included all infections – asymptomatic and symptomatic. Age was included as a categorical variable with the age-group ‘5–9 years’ was chosen as the reference group as the numbers in this group were large and allowed clearer presentation of the risks in other groups. Poisson and logistic regression models were evaluated and the best model for the data determined by comparing the Akaike information criterion (AIC). To correct for repeated measures per individual, robust standard errors were calculated with allowance for clustering to account for non-independence of observations. Observations with missing data were excluded from the analyses. Variance inflation factors were also calculated to assess multicollinearity among the covariates included in the model (Supplementary Table 2 ^28^). Probability values ( *p*) of less than 0.05 were considered statistically significant. All statistical analyses were carried out in R statistical software via RStudio version 1.1.463 ^32^.

## Results

### Demography

**Figure 2.**
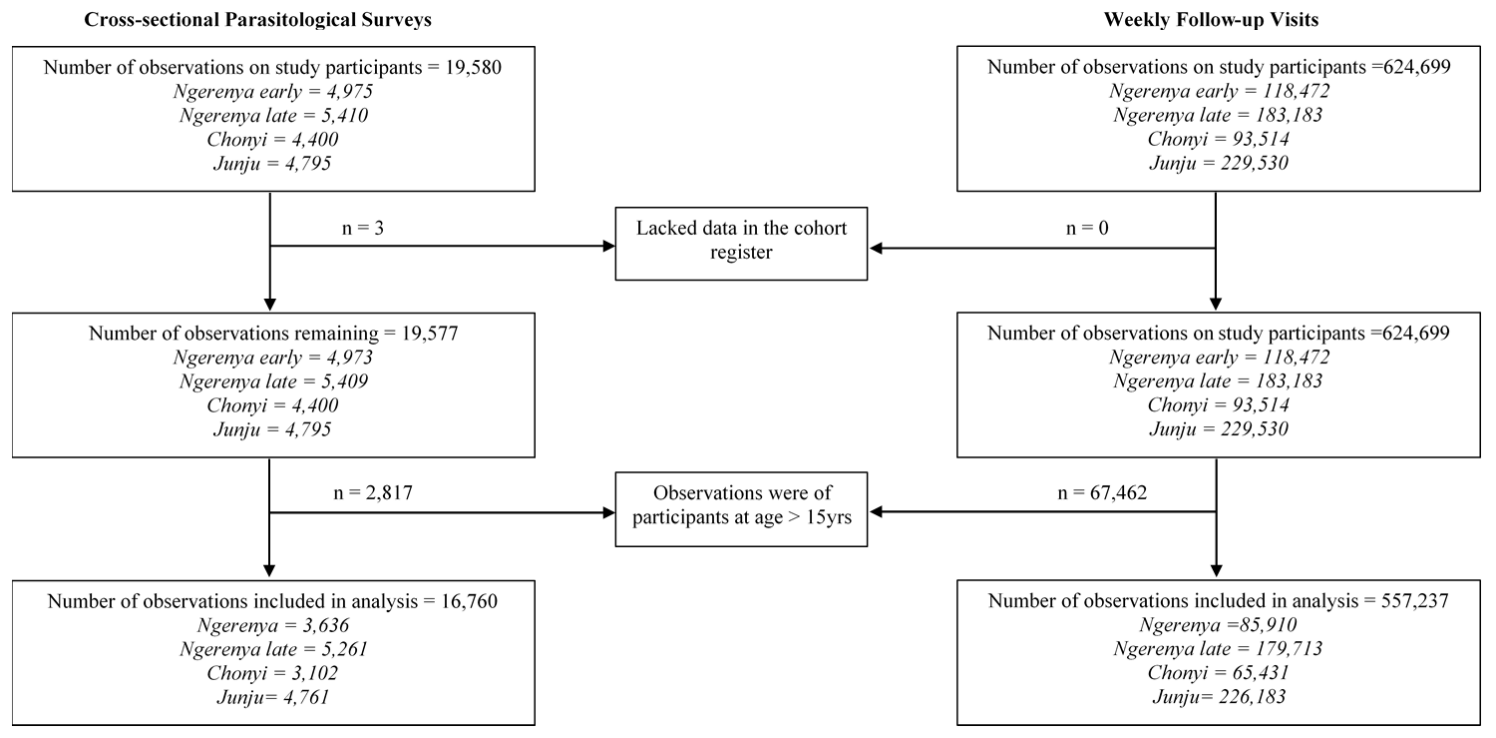
Flow diagram showing observations from cross-sectional surveys and weekly follow-up visits carried out on children recruited into the Kilifi Malaria Longitudinal Cohort. Reasons for exclusion at each step are also included.

**Figure 3.**
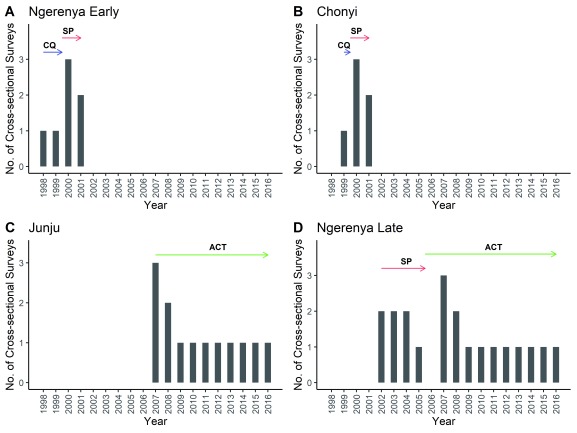
Summary of the number of cross-sectional surveys carried out per cohort and malaria drug in use per year for each cohort. Bar graphs showing the number of cross-sectional surveys and the year of the surveys carried out during the follow-up period included in this analysis. Arrows above the bar plots indicate the malaria drug in use for each year with blue lines denoting chloroquine (CQ), red lines denoting sulfadoxine-pyrimethamine (SP) and green lines denoting artemisinin combination therapies (ACT).

For the study, a total of 19,580 observations from 2,703 children ( Figure 2) derived from cross-sectional surveys carried out between 1998 and 2016 ( Figure 3) were considered for analysis. There were 3 study participants missing in the cohort registry, 2,817 observations were aged >15 years and therefore excluded from the main analysis leaving 16,760 observations from 2,223 study participants for the main analysis, translating to over 9,134 person-years of observation. A total of 557,237 observations from the weekly follow-up data were also used in the analysis. The demographic characteristics of study participants participating in the cross-sectional surveys are presented in Table 1, the characteristics of the study participants participating in the weekly follow-up visits are provided in Table 2.

**Table 1.** Demographic characteristics of study participants participating in the cross-sectional surveys.

Variable	Cohort			
	Ngerenya		Chonyi	Junju
	Early	Late		
Total number of observations from study participants	3636	5261	3102	4761
Total number of females (%)	1714 (47.1)	2417 (45.9)	1513 (48.8)	2391 (50.2)
Person-years of follow-up	882	4164	984	3104
Number per age group (%)				
<0.5 years	151 (4.2)	133 (2.5)	117 (3.8)	77 (1.6)
0.5–1 year	157 (4.3)	186 (3.5)	152 (4.9)	182 (3.8)
1–5 years	1199 (33.0)	1745 (33.2)	970 (31.2)	1577 (33.1)
5–9 years	1078 (29.6)	1900 (36.1)	957 (30.9)	1792 (37.6)
9–12 years	725 (19.9)	889 (16.9)	598 (19.3)	729 (15.3)
12–15 years	326 (9.0)	408 (7.8)	308 (9.9)	404 (8.5)
Total number of asexual parasite positive observations (%)	984 (27.1)	199 (3.8)	1183 (38.1)	850 (17.9)
Total number of gametocyte positive observations (%)	164 (4.5)	20 (0.4)	142 (4.6)	38 (0.8)
Total number of malaria episodes *	899	419	530	2941
Missing data (%)				
Gametocyte density	0	47 (0.9)	0	71 (1.5)
Asexual parasite density	0	34 (0.6)	0	69 (1.4)
Temperature	432 (11.9)	22 (0.4)	21 (0.7)	0

*Malaria episodes calculated from weekly follow-up data for all study participants who had complete data on gametocyte density.

**Table 2.** Demographic characteristics of the participants participating in the weekly follow-up visits.

Variable	Cohort			
	Ngerenya		Chonyi	Junju
	Early	Late		
Total number of observations from study participants	85910	179713	65431	226183
Total number of females (%)	41011 (47.7)	82995 (46.2)	31780 (48.6)	113122 (50.0)
Number per age group (%)				
<0.5 years	3612 (4.2)	5032 (2.8)	2714 (4.1)	4968 (2.2)
0.5–1 year	3818 (4.4)	6326 (3.5)	2937 (4.5)	7497 (3.3)
1–5 years	29211 (34.0)	57704 (32.1)	20434 (31.2)	76800 (34.0)
5–9 years	25393 (29.6)	63612 (35.4)	20074 (30.7)	76496 (33.8)
9–12 years	16577 (19.3)	32156 (17.9)	12693 (19.4)	37452 (16.6)
12–15 years	7299 (8.5)	14883 (8.3)	6579 (10.1)	22970 (10.2)
Total number of asexual parasite positive observations (%)	4114 (4.8)	1072 (0.6)	3015 (4.6)	5900 (2.6)
Total number of gametocyte positive observations (%)	179 (0.2)	73 (0.04)	180 (0.3)	69 (0.03)
Total number of malaria episodes	1055	349	605	3493

### Parasite prevalence and density over time

**Figure 4.**
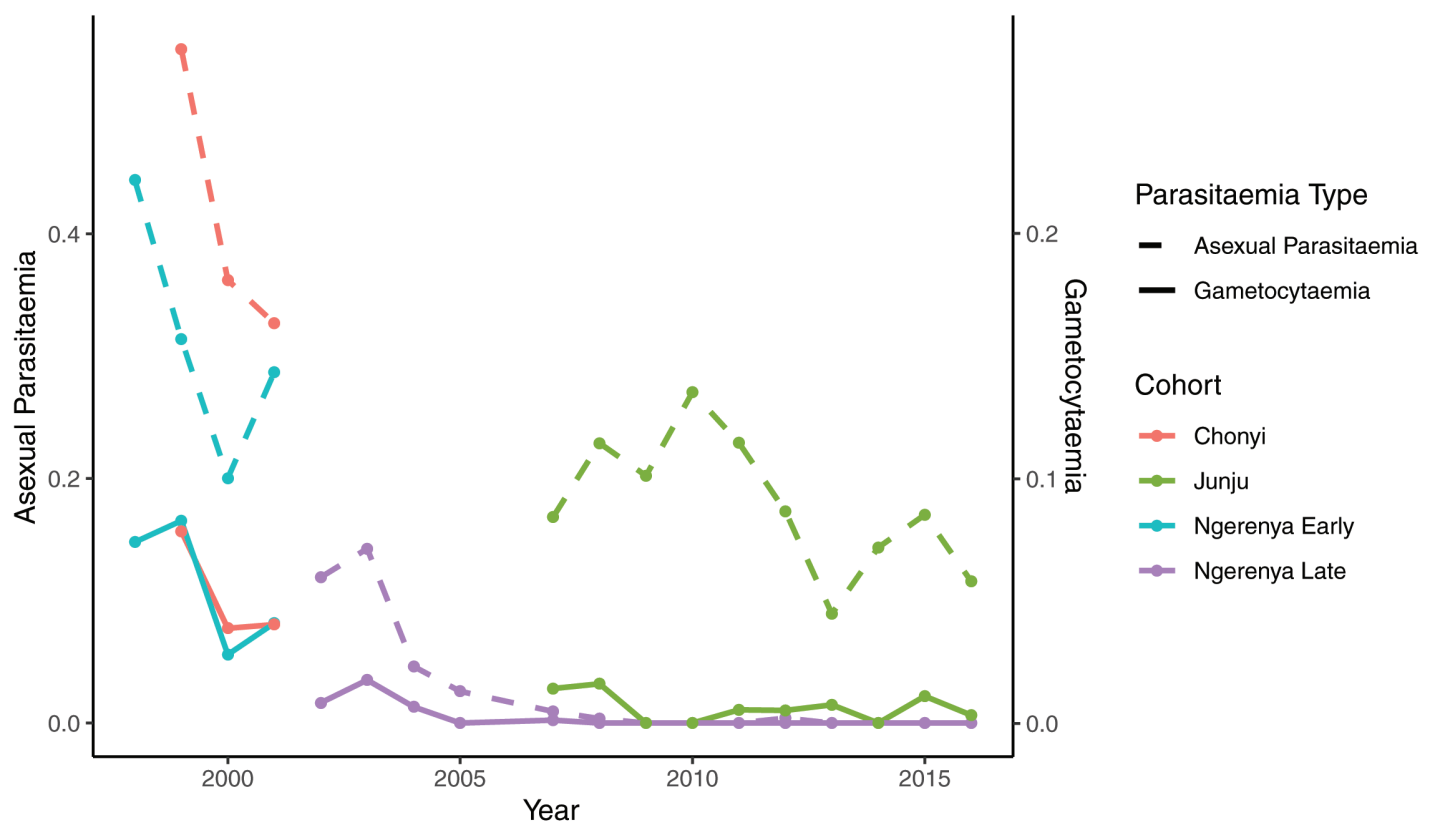
Parasite prevalence over time. Line plot showing the temporal variation in *P. falciparum* parasite prevalence as determined by microscopy. Overall Spearman’s rank correlation of the gametocyte and asexual parasite prevalence temporal variation was ρ=0.78 (p<0.0001).

**Figure 5.**
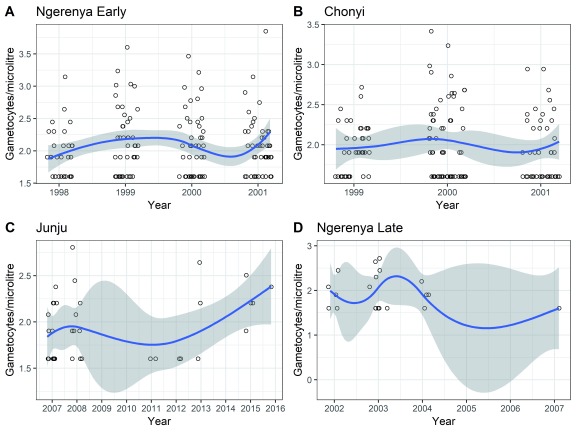
Scatter plots showing the change in gametocyte densities over time. Blue lines indicate mean values with the shaded grey areas representing 95% confidence intervals. ( **A**) Ngerenya early; ( **B**) Chonyi; ( **C**) Junju; and ( **D**) Ngerenya late.

**Figure 6.**
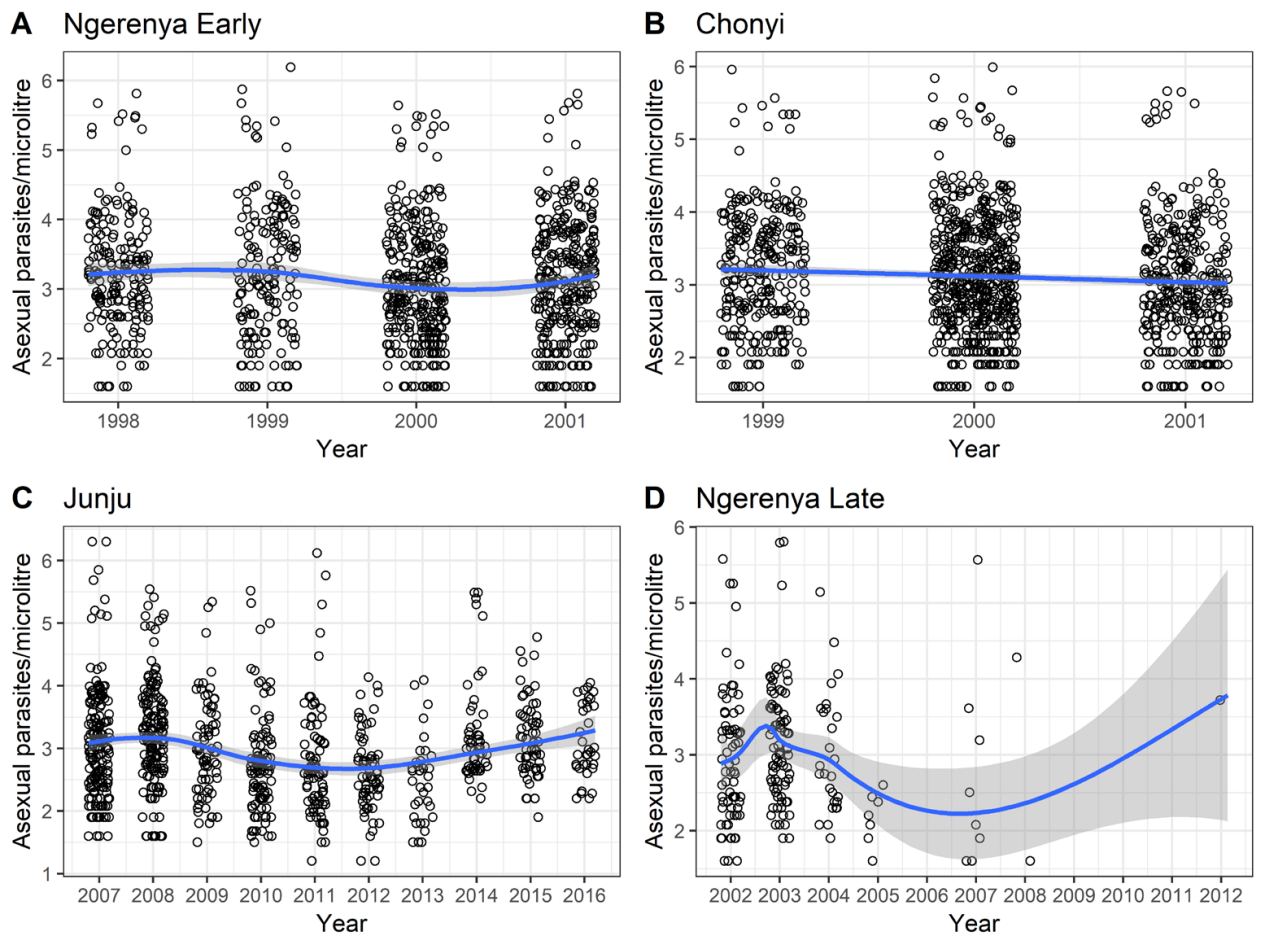
Scatter plots showing the change in asexual parasite densities over time. Blue lines indicate mean values with the shaded grey areas representing 95% confidence intervals ( **A**) Ngerenya early; ( **B**) Chonyi; ( **C**) Junju; and ( **D**) Ngerenya late.

Variation in the proportion with a positive blood film for *P. falciparum* asexual parasites or gametocytes over the period of follow-up was analysed for each of the cohorts ( Figure 4). In all the cohorts, the proportion positive for gametocytes was much lower than the proportion positive for asexual parasites. The overall correlation of gametocyte and asexual parasite prevalence over time is ρ = 0.78 (Spearman’s rank correlation, *p*<0.0001) indicating a paralleled decline in sexual and asexual parasitaemia. This was only true, however, in Ngerenya (both early and late transmission periods) and Chonyi. For Junju the temporal variation was more random, (the calculated correlation coefficient ρ = -0.09, *p* = 0.8) demonstrating the absence of a strong relationship between asexual and sexual parasite prevalence over time in this cohort. On the other hand, gametocyte and asexual parasite densities did not differ significantly over time in the cohorts ( Figure 5 and Figure 6).

**Figure 7.**
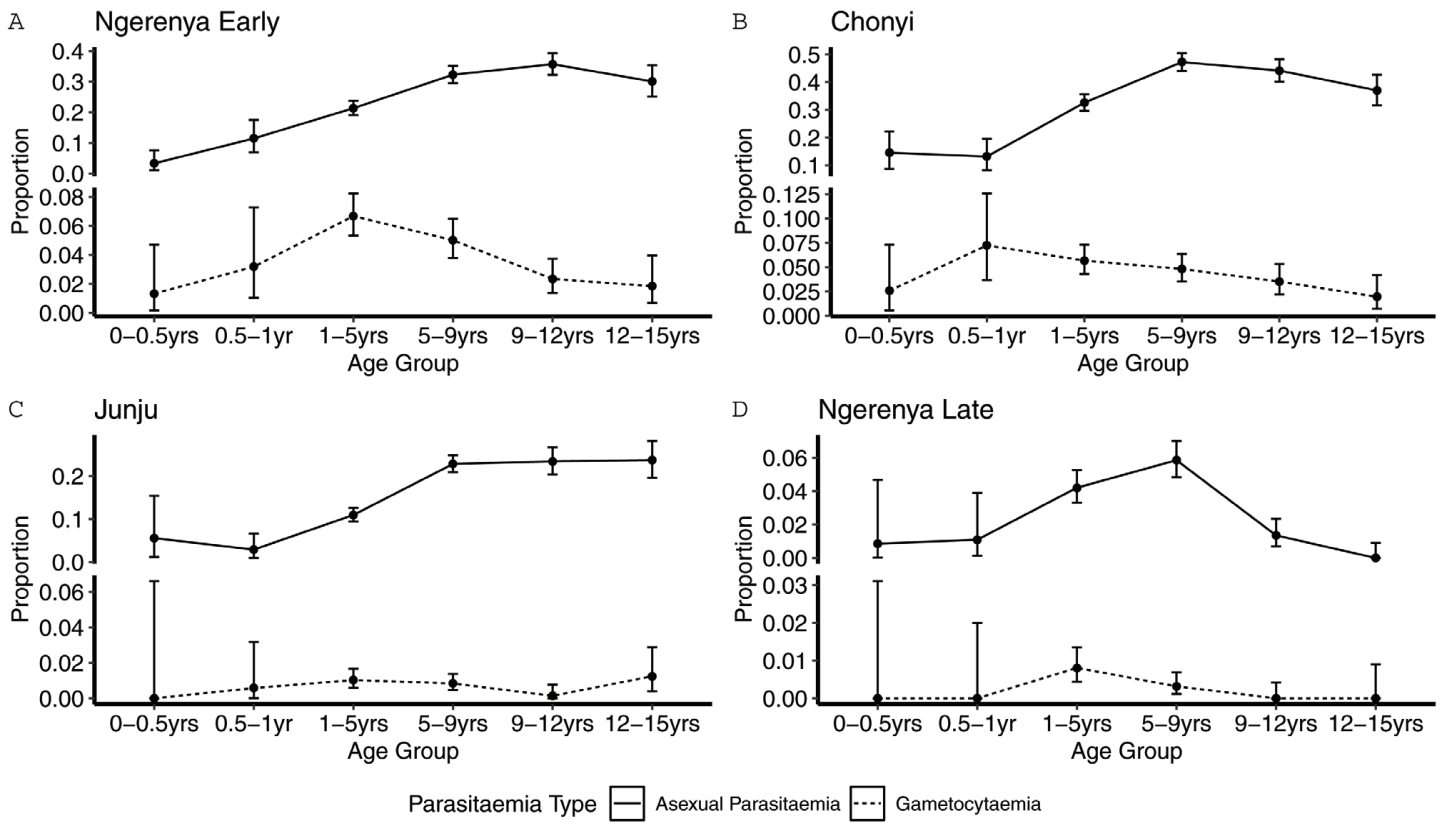
Parasite prevalence by age. Line graphs showing the variation in the proportion with gametocyte and asexual parasite positive blood films in the different age groups (0–0.5 years, 0.5–1 years, 1–5 years, 5–9 years, 9–12 years and 12–15 years) within the ( **A**) Ngerenya early; ( **B**) Chonyi; ( **C**) Junju; and ( **D**) Ngerenya late cohorts. The error bars indicate 95% confidence intervals.

To determine the association with age, an age-dependent variation in the proportion parasitaemic was also analysed in each cohort ( Figure 7). There was a peak prevalence of gametocytaemia among younger children in Chonyi (a high transmission setting) and Ngerenya early (a moderate to high transmission setting). For Junju (moderate to low transmission setting) and Ngerenya late (low transmission setting) sexual parasitaemia was less prevalent with no clear evidence of a peak. 

**Figure 8.**
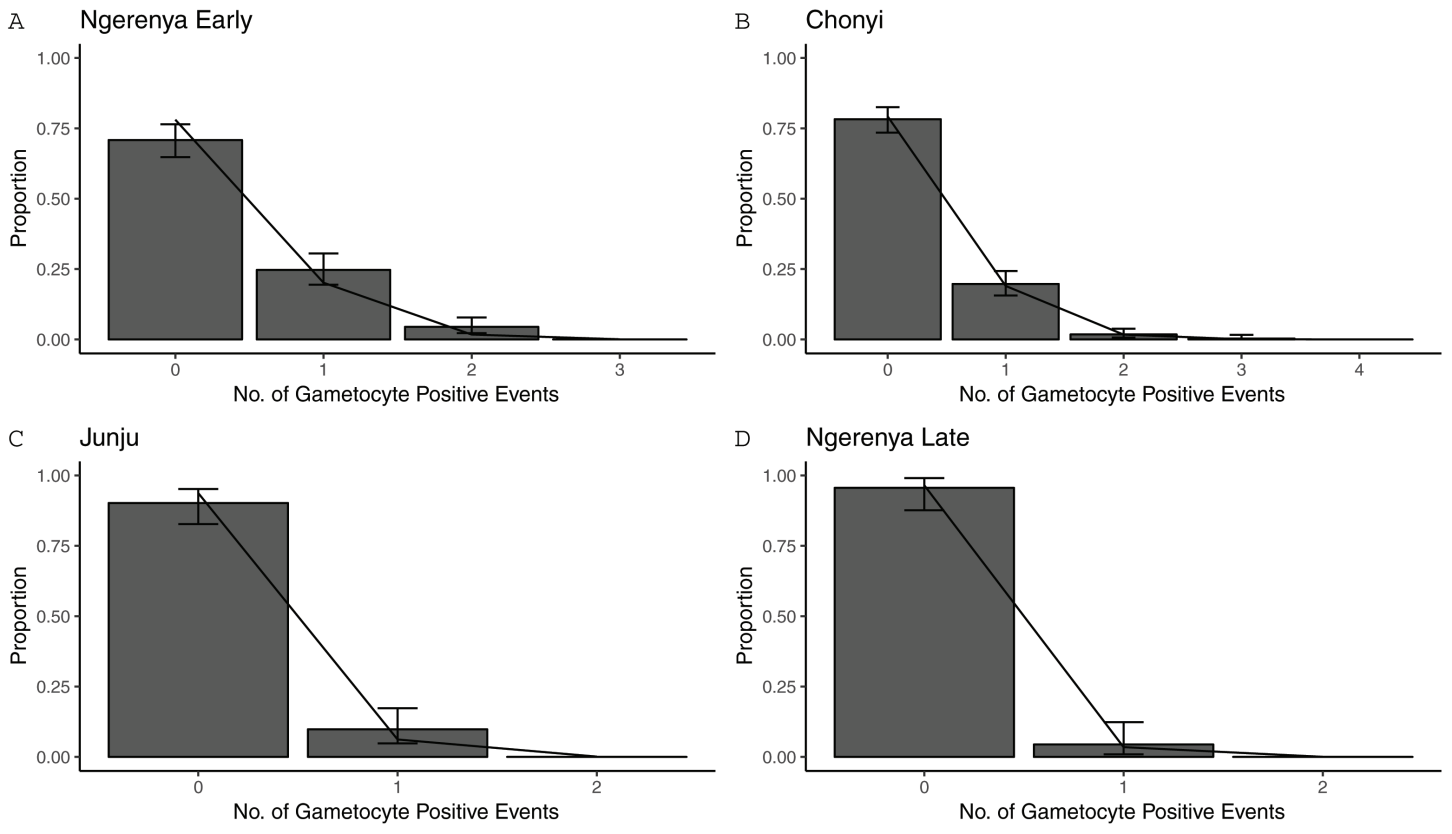
Distribution of individuals with multiple gametocyte positive blood films. Bar plot showing the number of individuals positive for gametocytes across all cohorts at the maximum blood film number for each cohort. Lines indicate the expected values for a binomial distribution. ( **A**) Ngerenya early; ( **B**) Chonyi; ( **C**) Junju; and ( **D**) Ngerenya late.

**Figure 9.**
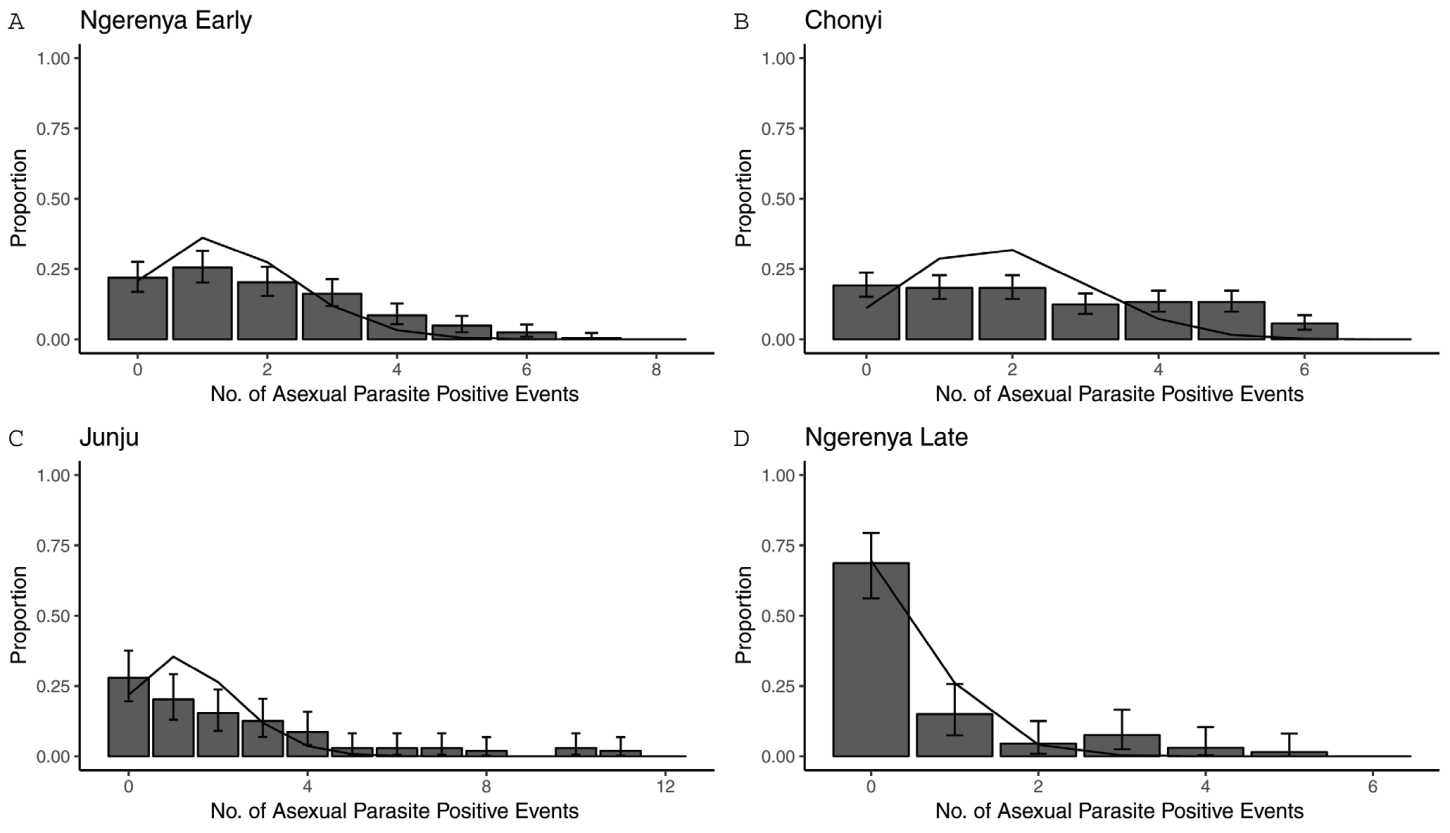
Distribution of individuals with multiple asexual parasite blood films. Bar plot showing the number of individuals positive for asexual parasites across all cohorts at the maximum blood film number for each cohort. Lines indicate the expected values for a binomial distribution. ( **A**) Ngerenya early; ( **B**) Chonyi; ( **C**) Junju; and ( **D**) Ngerenya late.

We further analysed the distribution of the number of parasite positive events per study participant for each of the cohorts. For each cohort the number of blood films taken (per individual) with the highest frequency was determined separately (Supplementary Figure 1 ^28^) and the analysis then restricted to individuals who had had the same number of blood films taken per cohort to avoid bias. The distribution of gametocyte positive events was approximately binomial ( Figure 8), while that of asexual parasite positive events was not ( Figure 9). This indicates that the frequencies of gametocyte carriage by individual approximates a binomial distribution, in contrast to the frequencies of asexual parasite carriage where we see a disproportionate number of individuals with a higher number of asexual parasite positive events than would be predicted.

### Factors predicting gametocyte positivity

We tested associations between the following covariates: asexual parasite positivity, age, cohort, number of malaria episodes and whether an individual was gametocyte positive or asexual parasite positive during the prior survey or had malaria episodes in the prior survey. Additionally, we compared using asexual parasitaemia as a binary variable (positive versus negative) and as a log-transformed continuous variable (with parasite negative individuals indicated as having one parasite per microlitre) (Supplementary Table 3 ^28^). We found a better fit for the model with asexual parasitaemia as a binary variable.

From the univariable analysis, having a blood film positive for asexual parasites, increased number of clinical malaria episodes in the survey period, and being positive for either asexual parasitaemia or gametocytes in the prior survey were all associated with increased odds of being gametocyte-positive ( Table 3). Residing in a lower-transmission setting (Junju and Ngerenya late) relative to high transmission setting (Chonyi) and older age, however, were associated with a decreased odds of being gametocyte positive. In the multivariable analysis all these factors remained significant independent predictors, except for asexual parasite positivity in the prior survey.

**Table 3.** Logistic regression model predicting gametocyte positivity.

Covariate	Univariable analysis			Multivariable analysis		
	Odds ratio	95% CI	*p* value	Odds ratio	95% CI	*p* value
Asexual parasite positive	6.70	5.38, 8.34	**<0.0001**	4.68	3.42, 6.39	**<0.0001**
Age group						
5 – 9 years	1.00	.	.	1.00	.	.
0 – 0.5 years	0.53	0.22, 1.31	0.1683	1.69	0.38, 7.48	0.4868
0.5 – 1 years	1.21	0.71, 2.08	0.4842	2.36	1.29, 4.33	**0.0054**
1 – 5 years	1.44	1.12, 1.85	**0.0041**	1.75	1.30, 2.36	**0.0002**
9 –12 years	0.62	0.43, 0.90	**0.0293**	0.62	0.41, 0.93	**0.0219**
12 – 15 years	0.55	0.32, 0.94	**0.0118**	0.67	0.37, 1.23	0.1961
Cohort						
Chonyi	1.00	.	.	1.00	.	.
Junju	0.17	0.12, 0.25	**<0.0001**	0.21	0.13, 0.34	**<0.0001**
Ngerenya early	0.98	0.77, 1.25	0.8991	1.16	0.87, 1.54	0.3240
Ngerenya late	0.08	0.05, 0.13	**<0.0001**	0.18	0.11, 0.32	**<0.0001**
Number of malaria episodes ^i^	1.27	1.17, 1.38	**<0.0001**	1.37	1.20, 1.56	**<0.0001**
Number of malaria episodes in the prior survey	1.04	0.89, 1.21	0.6268	0.91	0.74, 1.12	0.3722
Gametocyte positive in the prior survey	4.58	2.95, 7.13	**<0.0001**	2.00	1.25, 3.20	**0.0039**
Asexual parasite positive in the prior survey	2.16	1.68, 2.80	**<0.0001**	0.86	0.64, 1.16	0.3321

Log likelihood test for model including age as a covariate *p*<0.0001. Robust standard error estimation; Wald test *F* statistic - 36.17, *p*<0.0001. ^i ^the ‘number of malaria episodes’ is defined as the sum of the number of malaria episodes occurring in the period leading up to a cross-sectional survey. The *p* values in bold are statistically significant at significance level 0.05

Associations were consistent when the models were fitted separately for each cohort ( Table 4– Table 7), except that the number of clinical malaria episodes in the survey period had different associations in the different cohorts. The number of malaria episodes occurring in the period leading up to a cross-sectional survey were associated with increased odds of gametocyte positivity in Chonyi (OR 1.49, 95% CI 1.06-2.12, *p* = 0.0234), Ngerenya early (OR 1.68, 95% CI 1.34-2.12, *p* <0.0001) and Ngerenya late (OR 1.30, 95% CI 0.60-2.81, *p* = 0.5018). However, in Junju the number of malaria episodes was associated with reduced odds of gametocyte positivity (OR 0.73, 95% CI 0.40-1.33, *p* = 0.3037). We tested the interaction between the number of malaria episodes and cohort in a logistic regression and confirmed that the variation in effect of malaria episodes was statistically significant ( *p* = 0.0297) ( Table 8). Furthermore, noting that asexual parasitaemia was quantitatively more strongly associated with gametocytaemia in Junju than in other cohorts, we tested the interaction between being asexual parasite positive in the three cohorts. We found that relative to Chonyi, there was an observed increased odds of gametocyte positivity with asexual parasite positivity only in Junju ( *p* = 0.0007) and Ngerenya late ( *p* = 0.0008).

**Table 4.** Logistic regression model predicting gametocyte positivity using data from Ngerenya early cohort only.

Covariate	Univariable analysis			Multivariable analysis		
	Odds ratio	95% CI	*p* value	Odds ratio	95% CI	*p* value
Asexual parasite positive	3.16	2.29, 4.35	**<0.0001**	3.14	2.07, 4.78	**<0.0001**
Age group						
5–9 years	1.00	.	.	1.00	.	.
0–0.5 years	0.25	0.06, 1.04	0.0570	3.01	0.66, 13.80	0.1565
0.5–1 years	0.62	0.24, 1.59	0.3225	0.88	0.28, 2.78	0.8336
1–5 years	1.36	0.94, 1.95	0.1025	1.63	1.03, 2.57	**0.0363**
9–12 years	0.46	0.26, 0.80	**0.0057**	0.59	0.31, 1.13	0.1094
12–15 years	0.36	0.14, 0.94	**0.0361**	0.52	0.17, 1.56	0.2430
Number of malaria episodes ^i^	1.85	1.54, 2.22	**<0.0001**	1.68	1.34, 2.12	**<0.0001**
Number of malaria episodes in the prior survey	1.01	0.73, 1.39	0.9738	0.81	0.58, 1.13	0.2134
Gametocyte positive in the prior survey	2.23	1.20, 4.16	**0.0113**	1.75	0.85, 3.60	0.1266
Asexual parasite positive in the prior survey	1.12	0.75, 1.67	0.5731	0.82	0.51, 1.30	0.3935
Year	0.72	0.61, 0.86	**0.0002**	0.86	0.62, 1.19	0.3559

^i^ the ‘number of malaria episodes’ is defined as the sum of the number of malaria episodes occurring in the period leading up to a cross-sectional survey. Robust standard error estimation; Wald test *F* statistic - 8.84, *p*<0.0001. The *p* values in bold <0.05.

**Table 5.** Logistic regression model predicting gametocyte positivity using data from Chonyi cohort only.

Covariate	Univariable analysis			Multivariable analysis		
	Odds ratio	95% CI	*p* value	Odds ratio	95% CI	*p* value
Asexual parasite positive	2.96	2.07, 4.22	**<0.0001**	3.05	1.90, 4.88	**<0.0001**
Age group						
5 – 9 years	1.00	.	.	1.00	.	.
0 – 0.5 years	0.52	0.16, 1.70	0.2804	N/A	N/A	N/A
0.5 – 1 years	1.55	0.74, 3.21	0.2433	3.43	1.55, 7.57	**0.0023**
1 – 5 years	1.19	0.79, 1.79	0.4027	1.36	0.82, 2.27	0.2345
9 –12 years	0.72	0.43, 1.21	0.2168	0.94	0.51, 1.72	0.8390
12 – 15 years	0.39	0.17, 0.93	**0.0334**	0.61	0.24, 1.58	0.3070
Number of malaria episodes ^i^	2.01	1.55, 2.60	**<0.0001**	1.49	1.06, 2.12	**0.0234**
Number of malaria episodes in the prior survey	1.61	1.18, 2.21	**0.0029**	1.29	0.93, 1.81	0.1290
Gametocyte positive in the prior survey	2.26	1.06, 4.78	**0.0339**	1.84	0.88, 3.84	0.1052
Asexual parasite positive in the prior survey	1.22	0.81, 1.84	0.3442	0.91	0.59, 1.40	0.6725
Year	0.71	0.54, 0.93	**0.0117**	1.01	0.67, 1.54	0.9472

^i^ the ‘number of malaria episodes’ is defined as the sum of the number of malaria episodes occurring in the period leading up to a cross-sectional survey. Robust standard error estimation; Wald test *F* statistic - 189.17, *p*<0.0001. *P* values in bold <0.05. N/A - sample size insufficient for estimate (i.e. n<5).

**Table 6.** Logistic regression model predicting gametocyte positivity using data from Junju cohort only.

Covariate	Univariable analysis			Multivariable analysis		
	Odds ratio	95% CI	*p* value	Odds ratio	95% CI	*p* value
Asexual parasite positive	20.76	9.02, 47.77	**<0.0001**	18.52	6.78, 50.58	**<0.0001**
Age group						
5–9 years	1.00	.	.	1.00	.	.
0–0.5 years	N/A	N/A	N/A	N/A	N/A	N/A
0.5–1 years	0.69	0.09, 5.15	0.7150	5.80	0.79, 42.69	0.0842
1–5 years	1.23	0.58, 2.59	0.5858	1.92	0.81, 4.57	0.1380
9–12 years	0.16	0.54, 4.15	0.0809	0.20	0.03, 1.50	0.3757
12–15 years	1.49	0.02, 1.25	0.4431	2.02	0.43, 9.61	0.1184
Number of malaria episodes ^i^	0.67	0.39, 1.16	0.1546	0.73	0.40, 1.33	0.3037
Number of malaria episodes in the prior survey	0.92	0.59, 1.43	0.7066	1.12	0.70, 1.79	0.6406
Gametocyte positive in the prior survey	4.64	0.62, 34.84	0.1357	1.85	0.26, 13.19	0.5399
Asexual parasite positive in the prior survey	1.93	0.88, 4.23	0.0987	0.68	0.28, 1.61	0.3767
Year	0.86	0.75, 0.98	**0.0273**	0.96	0.79, 1.15	0.6349

^i^ the ‘number of malaria episodes’ is defined as the sum of the number of malaria episodes occurring in the period leading up to a cross-sectional survey. Robust standard error estimation; Wald test *F* statistic - 41.06, *p*<0.0001. *P* values in bold < 0.05. N/A - sample size insufficient for estimate (i.e. n<5).

**Table 7.** Logistic regression model predicting gametocyte positivity using data from Ngerenya late cohort only.

Covariate	Univariable analysis			Multivariable analysis		
	Odds ratio	95% CI	*p* value	Odds ratio	95% CI	*p* value
Asexual parasite positive	40.39	15.78, 103.38	**<0.0001**	22.07	5.70, 85.41	**<0.0001**
Age group						
5–9 years	1.00	.	.	1.00	.	.
0–0.5 years	N/A	N/A	N/A	N/A	N/A	N/A
0.5–1 years	N/A	N/A	N/A	N/A	N/A	N/A
1–5 years	2.56	0.97, 6.77	0.0584	2.69	0.89, 8.12	0.0787
9–12 years	N/A	N/A	N/A	N/A	N/A	N/A
12–15 years	N/A	N/A	N/A	N/A	N/A	N/A
Number of malaria episodes ^i^	3.25	2.21, 4.78	**<0.0001**	1.30	0.60, 2.81	0.5018
Number of malaria episodes in the prior survey	0.65	0.13, 3.17	0.5963	0.19	0.02, 1.63	0.1308
Gametocyte positive in the prior survey	18.01	4.35, 74.56	**<0.0001**	9.72	0.75, 125.70	0.0816
Asexual parasite positive in the prior survey	3.48	1.07, 11.34	**0.0384**	0.25	0.04, 1.71	0.1581
Year	0.59	0.49, 0.71	**<0.0001**	0.74	0.58, 0.94	**0.0129**

^i^ the ‘number of malaria episodes’ is defined as the sum of the number of malaria episodes occurring in the period leading up to a cross-sectional survey. Robust standard error estimation; Wald test *F* statistic - 522.09, *p*<0.0001 *P* values in bold < 0.05. N/A - sample size insufficient for estimate (i.e. n<5).

**Table 8.** Logistic regression model predicting gametocyte positivity with interaction analysis.

Covariate	Univariable analysis			Multivariable analysis		
	Odds ratio	95% CI	*p value*	Odds ratio	95% CI	*p value*
Asexual parasite positive	6.70	5.38, 8.34	**<0.0001**	3.25	2.09, 5.05	**<0.0001**
Age group						
5–9 years	1.00	.	.	1.00	.	.
0–0.5 years	0.53	0.22, 1.31	0.1683	1.60	0.38, 6.84	0.5226
0.5–1 years	1.21	0.71, 2.08	0.4842	2.19	1.20, 3.99	**0.0109**
1–5 years	1.44	1.12, 1.85	**0.0041**	1.71	1.26, 2.32	**0.0005**
9–12 years	0.62	0.43, 0.90	**0.0293**	0.67	0.44, 1.02	0.0610
12–15 years	0.55	0.32, 0.94	**0.0118**	0.72	0.40, 1.31	0.2783
Cohort						
Chonyi	1.00	.	.	1.00	.	.
Junju	0.17	0.12, 0.25	**<0.0001**	0.11	0.05, 0.27	**<0.0001**
Ngerenya early	0.98	0.77, 1.25	0.8991	1.02	0.67, 1.56	0.9149
Ngerenya late	0.08	0.05, 0.13	**<0.0001**	0.07	0.04, 0.15	**<0.0001**
Number of malaria episodes ^i^	1.27	1.17, 1.38	<0.0001	1.54	1.11, 2.12	**0.0088**
Number of malaria episodes in the prior survey	1.04	0.89, 1.21	0.6268	0.94	0.76, 1.18	0.6069
Gametocyte positive in the prior survey	4.58	2.95, 7.13	<0.0001	1.91	1.20, 3.05	**0.0067**
Asexual parasite positive in the prior survey	2.16	1.68, 2.80	**<0.0001**	0.81	0.61, 1.07	0.1364
Asexual parasite positive: Chonyi	1.00	.	.	1.00	.	.
Asexual parasite positive: Junju	7.02	2.84, 17.37	**<0.0001**	5.12	1.99, 13.22	**0.0007**
Asexual parasite positive: Ngerenya early	1.07	0.66, 1.73	0.3557	0.97	0.54, 1.72	0.9055
Asexual parasite positive: Ngerenya late	13.66	5.00, 37.31	**<0.0001**	9.62	2.56, 36.16	**0.0008**
Chonyi: Number of malaria episodes	1.00	.	.	1.00	.	.
Junju: Number of malaria episodes	0.33	0.18, 0.61	**0.0004**	0.49	0.25, 0.93	**0.0297**
Ngerenya early: Number of malaria episodes	0.92	0.67, 1.26	0.6055	1.11	0.76, 1.62	0.5965
Ngerenya late: Number of malaria episodes	1.62	1.02, 2.57	**0.0425**	1.04	0.46, 2.34	0.9233

^i^ the ‘number of malaria episodes’ is defined as the sum of the number of malaria episodes occurring in the period leading up to a cross-sectional survey. Robust standard error estimation; Wald test *F* statistic - 22.12, *p*<0.0001, *p* values in bold are statistically significant at significance level 0.05.

**Figure 10.**
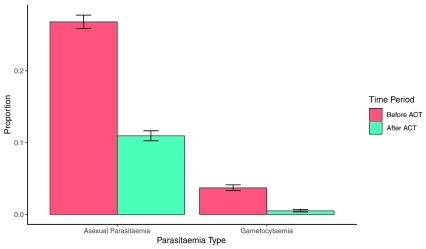
Prevalence of gametocytaemia and asexual parasitaemia before and after artemisinin combination therapy (ACT) introduction in all cohorts. Bar plot showing the proportion of study participants positive for gametocytes or asexual parasites before and after introduction of ACTs. Prevalence of gametocytaemia was 4% before ACTs and 0.5% after ACTs, while the prevalence of asexual parasitaemia was 27% and 11% respectively.

Owing to differential associations observed in the cohorts between malaria episodes and gametocyte positivity and the difference in follow-up period, particularly for Junju and Chonyi, we divided the dataset into two time-periods, before 2006 and after 2006. This marked the periods before and after the introduction of ACTs. There was a marked decline in gametocyte prevalence in the period after the introduction of ACTs ( Figure 10), dropping from approximately 4% to 0.5%. We adjusted for malaria episodes occurring within 28 days of the cross-sectional survey ( Table 9 and Table 10). We found that before 2006, the number of malaria episodes were associated with an increased risk of gametocyte positivity (OR 1.38, 95% CI 1.15-1.66, *p* = 0.0006) while malaria episodes occurring within 28 days of a cross-sectional survey were associated with an approximately threefold increased risk of gametocyte positivity (95% CI 1.85, 4.53, *p* <0.0001). On the other hand, after 2006 the number of malaria episodes a participant had and malaria episodes occurring within 28 days of a cross-sectional survey ceased to be predictors of gametocyte positivity (OR 0.68, 95% CI 0.39, 1.20, *p* = 0.1809 and OR 1.46, 95% CI 0.21-10.01, *p* = 0.7026, respectively).

**Table 9.** Logistic regression model predicting gametocyte positivity in observations recorded before 2006.

Covariate	Univariable analysis			Multivariable analysis		
	Odds ratio	95% CI	*p* value	Odds ratio	95% CI	*p* value
Asexual parasite positive	3.93	3.12, 4.95	**<0.0001**	3.49	2.56, 4.78	**<0.0001**
Age group						
5–9 years	1.00	.	.	1.00	.	.
0–0.5 years	0.37	0.15, 0.91	**0.0310**	1.63	0.39, 6.84	0.5038
0.5–1 years	1.06	0.60, 1.87	08350	1.95	1.05, 3.62	**0.0335**
1–5 years	1.31	1.00, 1.70	**0.0488**	1.63	1.19, 2.25	**0.0027**
9–12 years	0.72	0.50, 1.06	0.0937	0.75	0.48, 1.15	0.1883
12–15 years	0.50	0.26, 0.96	**0.0359**	0.58	0.28, 1.20	0.1409
Cohort						
Chonyi	1.00	.	.	1.00	.	.
Ngerenya early	0.98	0.77, 1.25	0.8991	1.06	0.80, 1.42	0.6698
Ngerenya late	0.20	0.12, 0.32	**<0.0001**	0.31	0.19, 0.53	**<0.0001**
Number of malaria episodes ^i^	1.92	1.67, 2.20	**<0.0001**	1.38	1.15, 1.66	**0.0006**
Number of malaria episodes in the prior survey	1.18	0.95, 1.47	0.1353	0.88	0.69, 1.12	0.3029
Gametocyte positive in the prior survey	2.76	1.75, 4.34	**<0.0001**	2.01	1.23, 3.29	**<0.0001**
Asexual parasite positive in the prior survey	1.40	1.07, 1.83	**0.0146**	0.86	0.63, 1.16	**0.0132**
Malaria episode within 28 days of survey						
No	1.00	.	.	1.00	.	.
Yes	5.60	4.03, 7.79	**<0.0001**	2.89	1.85, 4.53	**<0.0001**

^i^ the ‘number of malaria episodes’ is defined as the sum of the number of malaria episodes occurring in the period leading up to a cross-sectional survey. Robust standard error estimation; Wald test *F* statistic - 19.73 *p*<0.0001. *P* values in bold < 0.05.

**Table 10.** Logistic regression model predicting gametocyte positivity in observations recorded after 2006.

Covariate	Univariable analysis			Multivariable analysis		
	Odds ratio	95% CI	*p value*	Odds ratio	95% CI	*p value*
Asexual parasite positive	38.60	16.77, 88.81	**<0.0001**	23.73	7.45, 75.53	**<0.0001**
Age group						
5–9 years	1.00	.	.	1.00	.	.
0–0.5 years	N/A	N/A	N/A	N/A	N/A	N/A
0.5–1 years	0.74	0.10, 5.56	0.7689	6.31	0.89, 44.60	0.0650
1–5 years	1.37	0.66, 2.89	0.3944	2.17	0.91, 5.20	0.0807
9–12 years	0.12	0.02, 0.94	**0.0435**	0.18	0.02, 1.44	0.1060
12–15 years	1.18	0.42, 3.26	0.7545	1.65	0.55, 4.93	0.3738
Cohort						
Junju	1.00	.	.	1.00	.	.
Ngerenya late	0.04	0.01, 0.28	**0.0013**	0.16	0.02, 1.70	0.1293
Number of malaria episodes ^i^	0.91	0.59, 1.39	0.6493	0.68	0.39, 1.20	0.1809
Number of malaria episodes in the prior survey	1.16	0.83, 1.62	0.3815	1.09	0.71, 1.67	0.6970
Gametocyte positive in the prior survey	7.58	1.01, 56.90	**0.0489**	1.86	0.27, 13.02	0.5321
Asexual parasite positive in the prior survey	3.44	1.58, 7.49	**0.0019**	0.65	0.27, 1.54	0.3249
Malaria episode within 28 days of survey						
No	1.00	.	.	1.00	.	.
Yes	1.01	0.14, 7.43	0.994	1.46	0.21, 10.01	0.7026

^i^ the ‘number of malaria episodes’ is defined as the sum of the number of malaria episodes occurring in the period leading up to a cross-sectional survey. Robust standard error estimation; Wald test *F* statistic - 41.11, *p*<0.0001. *P* values in bold < 0.05. N/A - sample size insufficient for estimate (i.e. n<5).

Additionally, we looked at how gametocyte positivity in individuals who tested positive for asexual parasites varied with age, cohort, malaria episodes and parasitaemia (Supplementary Tables 4 and 5 ^28^). In this analysis, we found that being under 5 years of age and being gametocyte-positive in the prior survey were associated with an increased odds of gametocyte positivity, while residing in Junju was associated with a decreased odds of gametocyte positivity, consistent with the analysis in Table 2.

We also tested for associations between the genetic factors for sickle cell, α-thalassaemia, and blood group on gametocyte positivity in a subset of individuals for whom genotype data was available (Supplementary Table 6 ^28^). Heterozygosity (OR 0.92, 95% CI 0.66-1.27, *p* = 0.6170) and homozygosity (OR 0.70, 95% CI 0.43-1.15, *p* = 0.1561) for α-thalassaemia did not appear associated with gametocyte positivity. This was also true for sickle cell trait (OR 1.23, 95% CI 0.82-1.85, *p* = 0. 3082, Table 11). There were only nine children with sickle cell disease, hence we do not present an odds ratio for these children. We found that relative to AB and A blood groups, B and O blood groups were not associated with gametocyte carriage in this cohort (OR 1.34, 95% CI 0.64-2.79, *p* = 0.4419 and OR 1.03, 95% CI 0.57-1.86, *p* = 0.9294, respectively) ( Table 12).

**Table 11.** Logistic regression model predicting gametocyte positivity including sickle cell and α-thalassaemia genotype data.

Covariate	Univariable analysis			Multivariable analysis		
	Odds ratio	95% CI	*p value*	Odds ratio	95% CI	*p value*
Asexual parasite positive	8.24	6.34, 10.71	**<0.0001**	5.94	4.15, 8.48	**<0.0001**
Age group						
5–9 years	1.00	.	.	1.00	.	.
0–0.5 years	0.56	0.18, 1.79	0.3287	2.93	0.64, 13.46	0.1671
0.5–1 years	1.15	0.56, 2.36	0.7091	1.60	0.68, 3.78	0.2813
1–5 years	1.59	1.17, 2.17	**0.0030**	1.65	1.17, 2.33	**0.0044**
9–12 years	0.56	0.34, 0.91	**0.0195**	0.47	0.28, 0.81	**0.0059**
12–15 years	0.37	0.17, 0.81	**0.0127**	0.37	0.15, 0.90	**0.0292**
Cohort						
Chonyi	1.00	.	.	1.00	.	.
Junju	0.19	0.12, 0.29	**<0.0001**	0.21	0.12, 0.35	**<0.0001**
Ngerenya early	1.12	0.80, 1.56	0.5084	1.15	0.78, 1.69	0.4870
Ngerenya late	0.09	0.05, 0.15	**<0.0001**	0.19	0.10, 0.34	**<0.0001**
Number of malaria episodes ^i^	1.23	1.12, 1.35	**<0.0001**	1.30	1.12, 1.50	**0.0004**
Number of malaria episodes in the prior survey	1.02	0.85, 1.22	0.8374	0.88	0.68, 1.15	0.3507
Gametocyte positive in the prior survey	5.65	3.36, 9.50	**<0.0001**	1.99	1.11, 3.56	**0.0205**
Asexual parasite positive in the prior survey	2.22	1.62, 3.04	**<0.0001**	0.85	0.59, 1.22	0.3853
Sickle cell genotype						
Normal	1.00	.	.	1.00	.	.
Heterozygous	0.99	0.66, 1.48	0.9586	1.23	0.82, 1.85	0.3082
Homozygous	N/A	N/A	N/A	N/A	N/A	N/A
α-thalassaemia genotype						
Normal	1.00	.	.	1.00	.	.
Heterozygous	0.90	0.66, 1.22	0.4856	0.92	0.66, 1.27	0.6170
Homozygous	0.62	0.40, 0.97	**0.0351**	0.70	0.43, 1.15	0.1561

^i^ the ‘number of malaria episodes’ is defined as the sum of the number of malaria episodes occurring in the period leading up to a cross-sectional survey. Robust standard error estimation; Wald test *F* statistic - 56.98, *p*<0.0001. *P* values in bold < 0.05. N/A - sample size insufficient for estimate (i.e. n<5).

**Table 12.** Logistic regression model predicting gametocyte positivity including sickle cell, α-thalassaemia and blood group genotype data.

Covariate	Univariable Analysis			Multivariable Analysis		
	Odds ratio	95% CI	*p value*	Odds ratio	95% CI	*p value*
Asexual parasite positive	12.14	7.65, 19.27	**<0.0001**	10.97	6.11, 19.69	**<0.0001**
Age group						
5–9 years	1.00	.	.	1.00	.	.
0–0.5 years	1.06	0.14, 8.01	0.9584	3.18	0.26, 38.94	0.3657
0.5–1 years	1.80	0.52, 6.29	0.3541	1.14	0.22, 6.01	0.8740
1–5 years	3.11	1.69, 5.73	**0.0003**	1.85	0.91, 3.76	0.0895
9–12 years	0.14	0.02, 1.11	0.0623	0.15	0.02, 1.14	0.0662
12–15 years	1.07	0.35, 3.26	0.9106	1.07	0.34, 3.42	0.9029
Cohort						
Junju	1.00	.	.	1.00	.	.
Ngerenya early	8.51	5.00, 14.49	**<0.0001**	5.62	2.86, 11.04	**<0.0001**
Ngerenya late	0.36	0.18, 0.75	**0.0058**	0.64	0.30, 1.34	0.2387
Number of malaria episodes ^i^	1.22	1.06, 1.41	**0.0072**	1.09	0.86, 1.38	0.4660
Number of malaria episodes in the prior survey	1.06	0.81, 1.37	0.6787	0.85	0.55, 1.30	0.4494
Gametocyte positive in the prior survey	9.01	3.40, 23.88	**<0.0001**	2.38	0.81, 6.98	0.1142
Asexual parasite positive in the prior survey	2.00	1.08, 3.69	**0.0272**	0.64	0.32, 1.30	0.2189
Sickle cell genotype						
Normal	1.00	.	.	1.00	.	.
Heterozygous	0.86	0.42, 1.77	0.6827	1.07	0.51, 2.24	0.8560
Homozygous	N/A	N/A	N/A	N/A	N/A	N/A
α-thalassaemia genotype						
Normal	1.00	.	.	1.00	.	.
Heterozygous	1.12	0.65, 1.93	0.6865	0.99	0.57, 1.73	0.9811
Homozygous	0.45	0.17, 1.22	0.1165	0.45	0.16, 1.26	0.1299
ABO blood group						
A and AB	1.00	.	.	1.00	.	.
B	1.22	0.58, 2.57	0.5970	1.34	0.64, 2.79	0.4419
O	1.06	0.60, 1.88	0.8446	1.03	0.57, 1.86	0.9294

^i^ the ‘number of malaria episodes’ is defined as the sum of the number of malaria episodes occurring in the period leading up to a cross-sectional survey. Robust standard error estimation; Wald test *F* statistic - 31.67, *p*<0.0001. *P* values in bold <0.05. N/A - sample size insufficient for estimate (i.e. n<5).

## Discussion

The analysis aimed to describe gametocyte prevalence and distribution over time and varying transmission intensities, and to identify factors associated with gametocyte carriage in three cohorts of children in Kilifi maintained for 3, 12 and 19 years, respectively, in which individual follow-up ran to a maximum of 15 years of age. Identification of these factors could then possibly serve as predictive features that would allow for targeted application of transmission-reducing interventions ^11^. The three cohorts were in sublocations within Kilifi County (i.e. Ngerenya, Chonyi and Junju) that represent a low, a mid to low, and a high transmission setting. For the purposes of this analysis, however, Ngerenya was subdivided into Ngerenya early (a period of moderate to high transmission) and Ngerenya late (a period of low transmission).

In the Ngerenya early, Chonyi, and Ngerenya late cohorts, a trend towards lower gametocyte and asexual parasite prevalence over time was observed, but there was no clear trend in Junju. Malaria transmission has been on the decline on the Kenyan coast since 1998, as evidenced by a decrease in parasite prevalence and paediatric malaria admission cases. A resurgence in malaria transmission has been described, however, following a nadir in 2009/2010 ^33, 34^. Heterogeneity of transmission with hotspots of malaria has been described ^35^, and transmission has persisted in Junju, whilst in Ngerenya transmission has remained either non-existent or low in some parts. Previous literature has demonstrated reductions in the prevalence of gametocytaemia with increasing age in higher-transmission areas ^11, 17, 19, 31, 36, 37^, consistent with the pattern we here describe in the higher transmission cohorts. Similarly, results obtained in previous studies are confirmed by this analysis, that the likelihood of gametocytaemia increases in the presence of asymptomatic asexual parasitaemia ^6, 17, 31^. Furthermore, it is expected that the prevalence of gametocytaemia would fall in the community as the prevalence of asymptomatic parasitaemia falls ^38^. Moreover, prior episodes of clinical malaria are a well-documented source of gametocytaemia ^6, 11^. The findings in Junju are therefore unexpected: gametocyte prevalence was disproportionately lower compared with the prevalence of asexual parasitaemia ( Figure 4), and malaria episodes occurring in the period leading up to a cross-sectional survey were modestly protective rather than a risk factor for gametocytaemia ( Table 6). We speculate that a change in anti-malarial drug policy might have caused this variation in effect.

Based on national guidelines, treatment of malaria in Kenya was with CQ from the 1970s to 1999 before being replaced by SP that was used until late 2006 when it was replaced by ACTs ^33^. Both CQ and SP have been associated with increased gametocyte carriage post-treatment ^14, 36, 39^ and were in use in Ngerenya early and Chonyi for the treatment of malaria, possibly leading to a higher than expected gametocyte prevalence. On the other hand, in Junju, treatment for malaria during the period of follow-up included in this study was with ACTs, in particular the combination of artemether-lumefantrine, that has been described to reduce post-treatment gametocyte carriage ^4, 6, 40^, which may also explain the much lower gametocyte prevalence in Junju, and furthermore also explain the lack of association between prior episodes of clinical malaria and subsequent gametocytaemia.

Antimalarials have been used in various ways to control malaria. Mass drug administration has been effective in clearing gametocytes and reducing subsequent transmission intensity ^41^. Screen and treat has been proposed to avoid treating uninfected participants in mass drug administration, but has not been efficacious in field trials ^42^. In this study, we demonstrate that providing ACTs to the children with acute febrile malaria was associated with a cohort-wide reduction in the prevalence of gametocytaemia, and further evidence linking this effect to ACTs is the expected association between prior episodes of acute febrile malaria and gametocyte carriage in cohorts prior to ACT use, but the absence of this association after ACTs were introduced.

We acknowledge that Junju monitoring data is only available post ACT introduction and therefore we cannot analyse parasite prevalence pre- and post-ACT introduction in this cohort. Additionally, while monitoring data for Ngerenya spans pre- and post-ACT introduction, parasite prevalence is infrequent in Ngerenya after 2006 and thus we do not have enough power to detect the changing association between drug regimen and gametocyte prevalence. However, as Junju and Chonyi are located close to each other ( Figure 1) and have similar populations ^22^, and malaria parasites are likely to be mixed over this geographical space ^43^, it seems more likely that ACT use explains the changing epidemiological patterns rather than an ecological difference between the settings.

Gametocyte carriage depended more strongly on asexual parasite positivity at lower transmission intensities (Junju and Ngerenya late) than at higher transmission intensities (Ngerenya early and Chonyi). When gametocytes are seen in the absence of asexual parasitaemia by microscopy, it may be that asexual parasitaemia is present but below the threshold detectable microscopically ^44^. We hypothesise that since immunity to malaria develops more slowly at lower transmission settings ^45^, individuals will tend to have higher parasite densities that are more readily detected by microscopy and linked with an increased likelihood of gametocytaemia ^6, 17, 31^.

There was an indication for certain individuals being at a greater risk of gametocyte carriage, as being gametocyte-positive in the previous year predisposed a subject to gametocyte carriage. This has been described in Senegal, where Grange *et al.* (2015) identified hotspots of gametocyte carriage and these were associated with active malaria transmission ^11^. However, gametocytaemia appeared to follow a binomial distribution in contrast to the negative binomial distribution seen for asexual parasitaemia, suggestive that gametocyte carriage is more evenly distributed in the population compared with asexual parasite carriage, where certain individuals are at considerably greater susceptibility to [re-]infection due to host-related factors ^25^ ( Figure 8 and Figure 9).

Genetic polymorphisms known to be protective against severe malaria, such as B and O blood groups, sickle cell trait and α-thalassemia, were associated with increased gametocyte carriage in previous studies ^11, 12, 25, 46, 47^. However, in our dataset we were not able to replicate this finding.

A limitation of this study is that we did not study sub-microscopic infection. Sub-microscopic gametocyte carriage is common in malaria endemic areas and has been shown to contribute to malaria transmission ^48, 49^. We have previously shown that 45–75% of all mosquito infections result from parasite levels below the detection threshold of microscopy ^49^. Interestingly, this previous study by Goncalves *et al.* ^49^ and other studies ^16, 18^ showed that gametocyte prevalence was highest in 5–15-year-olds in comparison to their younger and older counterparts. This may reflect the lower parasite densities in these children owing to a more developed immune system that would be undetectable microscopically. This also indicates that transmission-reducing interventions may need to target more than just <5-year-olds to be effective. Therefore, employing the use of high-quality research-grade microscopy or quantitative PCR would be most beneficial in epidemiological studies aimed at identifying the infectious reservoir ^50^.

Another limitation of our study is that the microscopy protocol used in these cohorts is primarily for assessing asexual parasite carriage and therefore the number of blood films examined varied depending on the asexual parasite density. More fields were examined for asexual parasite negative blood films, which increased the chances of detecting gametocytes in these asexual parasite negative blood films. We observed, however, that more gametocytes were detected in blood films from individuals who were asexual parasite-positive, and furthermore that more gametocytes were detected at higher asexual parasite densities. Furthermore, any bias resulting from missing low-density gametocytaemia would be consistent across age, time, and other factors since the protocol has not been varied during the period of study, and we adjusted for asexual parasites in the multivariable models, and noted associations with gametocytaemia as reported in previous studies. We therefore do not believe this bias was responsible for the associations between covariates and risk of gametocytaemia seen in our study. 

## Conclusion

In summary, our analyses have confirmed the importance of age, transmission intensity and previous malaria episodes as predictors of microscopically detectable gametocyte carriage. The analyses including three different cohorts over 19 years of follow-up and varying transmission intensities allow a clear demonstration of the independence and interactions of these factors. These could serve as potential indicators of populations that contribute disproportionately to the infectious reservoir and where malaria transmission-blocking interventions could be prioritised. However, to improve characterisation of the infectious reservoir, epidemiological studies combining molecular tools for parasite detection together with assays to measure infectiousness to mosquitoes across all age groups and varied transmission settings will be required.

Our data also suggest that the introduction of ACTs, particularly the highly effective artemether/lumefantrine, may have had a substantial effect on gametocyte carriage among a cohort of children followed up actively both weekly and at cross-sectional surveys, disrupting the link between malaria episodes and subsequent gametocyte carriage. Based on the impact on gametocyte carriage, we infer a role for ACTs targeted used in febrile malaria cases as potentially impacting malaria transmission.

## Data availability

### Underlying data

Harvard Dataverse: Kilifi Malaria Longitudinal Cohort cross-sectional survey and weekly follow-up surveillance data for the estimation of parasite prevalence and factors associated with gametocyte carriage. https://doi.org/10.7910/DVN/18QB3V ^28^.

This project contains the following underlying data:

- csbleed_summary.tab (list of the number of cross-sectional surveys carried out each yeatr for each cohort).
- Datasets.zip (zipped package containing all datasets).
- imm_csbleed_data.tab (data from all cross-sectional surveys carried out between 1998 and 2016 for all participants, including cohort information, age, sex, blood group, study and participant ID number, asexual and sexual parasite density, body temperature and date of survey).
- imm_weekly_fu_overall.tab (data from weekly follow-up visits between 1998 and 2016, containing information on the same variables as the above dataset).
- sickle.thal data.tab (data on sickle cell and α-thalassaemia genotype of each participant).

The above raw data that support the findings of this manuscript are under restricted access and available through the KEMRI-Wellcome Trust Research Programme Data Governance Committee if the use of the data is complaint with the consent provided by the participants. Details of the criteria can be found in the KEMRI-Wellcome data sharing guidelines ( https://kemri-wellcome.org/about-us/#ChildVerticalTab_15). Requests for the data can be made to the Data Governance Committee ( dgc@kemri-wellcome.org) through the corresponding author.

### Extended data

Harvard Dataverse: Kilifi Malaria Longitudinal Cohort cross-sectional survey and weekly follow-up surveillance data for the estimation of parasite prevalence and factors associated with gametocyte carriage. https://doi.org/10.7910/DVN/18QB3V ^28^.

This project contains the following extended data:

- Supplementary figure 1_No. of Cross-sectional surveys attended
- Supplementary figure 1 legend
- Supplementary tables
- Kilifi_Malaria_Longitudinal_Cohort_Codebook KMLC
- Kilifi_Malaria_Longitudinal_Cohort_Data Readme
- Age and parasite prevalence.R
- Chi-square analysis.R
- Cross-sectional survey summary.R
- Gametocyte positivity model - Recent episodes of malaria.R
- Gametocyte positivity models.R
- Parasitaemia over time.R
- Parasite density over time.R
- Parasite prevalence by ACT period.R
- Summary of parasite positive events.R
- Summary of the csbleeds per year per cohort.R (csbleeds - cross-sectional surveys)
- Summary Statistics - csbleed.R (csbleeds - cross-sectional surveys)
- Summary Statistics - malaria episodes.R
- Summary Statistics - wfu.R (wfu - weekly follow-up)

Data are available under the terms of the Creative Commons Attribution 4.0 International license (CC-BY 4.0).
