# Supplementary material for: Gametocyte carriage in an era of changing malaria epidemiology: A 19-year analysis of a malaria longitudinal cohort
Source: Wellcome Open Res. 2019 May 28;4:66. Originally published 2019 Apr 5. [Version 2] doi: 10.12688/wellcomeopenres.15186.2 (PMC6557001; doi:10.12688/wellcomeopenres.15186.2)
Supplement: Peer review history

# Peer review: Reviewer response for version 2

**DOI:** 10.21956/wellcomeopenres.16700.r35627

**Published:** 2019-06-04

**Authors:** Katharine R. Trenholme

**Affiliations:** 1 Malaria Biology Laboratory, QIMR Berghofer Medical Research Institute, Herston, Australia

I am happy that the authors have addressed all points raised.

I have read this submission. I believe that I have an appropriate level of expertise to confirm that it is of an acceptable scientific standard.

# Peer review: Reviewer response for version 2

**DOI:** 10.21956/wellcomeopenres.16700.r35626

**Published:** 2019-05-28

**Authors:** Anna Rosanas-Urgell

**Affiliations:** 1 Department of Biomedical Sciences, Institute of Tropical Medicine Antwerp, Antwerp, Belgium

The authors have addressed appropriately all concerns.

I have read this submission. I believe that I have an appropriate level of expertise to confirm that it is of an acceptable scientific standard.

# Peer review: Reviewer response for version 1

**DOI:** 10.21956/wellcomeopenres.16568.r35236

**Published:** 2019-04-30

**Authors:** Katharine R. Trenholme

**Affiliations:** 1 Malaria Biology Laboratory, QIMR Berghofer Medical Research Institute, Herston, Australia

Muthui *et al* have used data collected between 1998 and 2016 to describe the distribution and prevalence of *P. falciparum* gametocytaemia in children from 3 regions of Kalifi which were chosen based on malaria transmission intensity.

 Thick and thin blood films were collected from children at cross sectional surveys and weekly follow ups over a 19 year period and asexual parasite and gametocyte densities were determined by microscopy.

 The aim of the study is to identify potential risk factors for gametocyte carriage with the view of identifying target populations for transmission blocking/reducing interventions. The analysis is largely descriptive and the conclusions reached are logical based on the data and findings.

 The manuscript is generally well written and the data clearly presented, however, a couple of points would benefit from additional clarification

- **Tables 1 and 2.** Describe the demographic characteristics of the study population, but it is not clear which data is included in which table. The footnote to Table 1 “malaria episodes calculated from weekly follow-up data for all study participants who had complete data on gametocyte density” is also confusing.
- **Under case detection.** “Treatment was freely provided…based on the Government of Kenya national guidelines for treatment of malaria” the drugs actually used are only described in the discussion (paragraph 3). It would be helpful to see this displayed visually on a figure (perhaps added to Figure 4). At the very least I suggest visually indicating the time when ACT was introduced.
- **Figure 10.** Prevalence of gametocytaemia and asexual parasitaemia before and after ACT introduction in all cohorts. But, Ngerenya is the only site for which data covering all 19 years is available. Chonyi (high transmission) was only sampled prior to 2006 when ACT was introduced and Junju (moderate transmission) was only sampled after 2006. Given this intermittent sampling of sites with different transmission intensities, can justification for inclusion of all data be included in the manuscript?

I have read this submission. I believe that I have an appropriate level of expertise to confirm that it is of an acceptable scientific standard, however I have significant reservations, as outlined above.

# Response

**Published:** 2019-05-19

**Authors:** Michelle Muthui

**Affiliations:** KEMRI-Wellcome Trust Research Programme, Kenya

We would like to sincerely thank you for taking the time to read our manuscript and provide constructive feedback. Please find our responses to your comments below:

- *Tables 1 and 2. Describe the demographic characteristics of the study population, but it is not clear which data is included in which table. The footnote to Table 1 “malaria episodes calculated from weekly follow-up data for all study participants who had complete data on gametocyte density” is also confusing.*

The data in Table 1 describes the data from the cross-sectional surveys while the data in Table 2 describes the data from the weekly follow-up visits. We agree that the table legends may not be as clear and have therefore renamed the title of Table 1 to read:

‘Table 1. Demographic characteristics of study participants participating in the cross-sectional surveys’.

The number of malaria episodes was calculated from the weekly follow-up data for the period leading up to each survey and then this figure assigned to the respective cross-sectional survey. This is what we present as the number of malaria episodes in Table 1.

- *Under case detection. “Treatment was freely provided…based on the Government of Kenya national guidelines for treatment of malaria” the drugs actually used are only described in the discussion (paragraph 3). It would be helpful to see this displayed visually on a figure (perhaps added to Figure 4). At the very least I suggest visually indicating the time when ACT was introduced.*

We agree that it would be useful to show the timelines when the different drug regimens were in use on a graph and have therefore added this to Figure 3 that also shows the number of cross-sectional surveys carried out in a year per cohort.

- *Figure 10. Prevalence of gametocytaemia and asexual parasitaemia before and after ACT introduction in all cohorts. But, Ngerenya is the only site for which data covering all 19 years is available. Chonyi (high transmission) was only sampled prior to 2006 when ACT was introduced and Junju (moderate transmission) was only sampled after 2006. Given this intermittent sampling of sites with different transmission intensities, can justification for inclusion of all data be included in the manuscript?*

We agree that the three cohorts have been followed up for different lengths of time and over different years. However, our primary objective was to describe parasite prevalence over time and varied transmission intensity and describe factors predictive of gametocyte carriage. Including the three cohorts observed under differing drug regimens and over different years reflects changes in malaria transmission would enable us to tease out the effects of changing drug regimen on the prevalence of gametocytaemia overall.

We do acknowledge, however, that the monitoring data for Junju is only available post-ACT introduction and therefore we cannot be certain that the changing epidemiology of parasite prevalence is due to the use of ACTs. Also, as hardly any parasites were detected in Ngerenya after 2006, we do not have sufficient power to detect a difference in parasite prevalence pre- and post-ACT introduction. In mitigation against this Junju and Chonyi are located close to each other with similar populations, and the malaria parasite populations are likely to be mixed over this geographical space. Therefore, it seems more likely that ACTs are the explanation for the changing epidemiological patterns rather than an ecological difference in the settings. We have added a paragraph in the discussion on page 17 after the second paragraph to explain this as below:

‘We acknowledge that Junju monitoring data is only available post ACT introduction and therefore we cannot analyse parasite prevalence pre- and post-ACT introduction in this cohort. Additionally, while monitoring data for Ngerenya spans pre- and post-ACT introduction, parasite prevalence is infrequent in Ngerenya after 2006 and thus we do not have enough power to detect the changing association between drug regimen and gametocyte prevalence. However, as Junju and Chonyi are located close to each other (Figure 1) and have similar populations, ^22^and malaria parasites are likely to be mixed over this geographical space, it seems more likely that ACT use explains the changing epidemiological patterns rather than an ecological difference between the settings.’

# Peer review: Reviewer response for version 1

**DOI:** 10.21956/wellcomeopenres.16568.r35372

**Published:** 2019-04-24

**Authors:** Anna Rosanas-Urgell

**Affiliations:** 1 Department of Biomedical Sciences, Institute of Tropical Medicine Antwerp, Antwerp, Belgium

The results presented in the manuscript are scientifically valid. The authors analyze 19 years of data on asexual/gametocyte carriage and predictor factors for gametocyte positivity based on light microscopy data. Although conclusions are clear and supported by the data presented, it was difficult to follow the definitions of the variables used and which data defined them. This could be improved by defining all variables in the 'Case definitions' section and using the same terms consistently through the text and tables. More detail is given below.

**Methods/Design:**

 3 cohorts with weekly follow up of clinical cases (febrile cases) + 1 annual survey for asymptomatic infections.

 Cross-sectionals surveys – numbers per community and year described in a figure.

- Is the 1 annual survey for asymptomatic infections mentioned in the methods section considered a survey (thus included in Fig 3 and supplementary Table 1)? It was not clear how this data was used.
- Through the text and in the tables the authors use different terms such as number of malaria episodes, current and previous survey, prior survey. Some of these terms are confusing. For example:

1. "Number of malaria episodes occurring between the current survey and the prior survey were then summed up and analyzed as the number of episodes occurring in the current survey."
  Is this variable defined by only weekly follow up data or also cross sectional surveys?
2. "Previous survey period interval between the preceding survey and the survey that came before."
  Is this using both cross-sectional and weekly follow up data? (Definition is found on page 9 of results).

Asexual parasite positive. Is it all infections or asymptomatic infections only?

 Malaria episode within 28 days of survey, is this variable the same than recent malaria episodes used in the text

 Study population: There are two tables presenting characteristics of the study population. Does Table 1 include all participants (both cohorts and cross sectionals) or does it include only cross-sectional? Or are cross-sectional study participants a sub-group of the cohort participants?

 While in the text the authors state “The demographic characteristics of study participants participating in the cross-sectional surveys are presented in Table 1, the characteristics of the study participants participating in the weekly follow-up visits are provided in Table 2”, Table 1 footer defines malaria episodes as calculated from weekly follow-up data and does not include cross-sectional in the title. This is confusing.

 Laboratory investigations: Describes an external and internal quality control of slides. Since the study covers 19 years, is this assurance system covering the full period? What was the result?

**Results:**

 (Page 9) "Prior clinical malaria episodes were associated with increased odds of gametocyte positivity in Chonyi (OR 1.49, 95% CI 1.06-2.12, *p* = 0.0234), Ngerenya early (OR 1.68, 95% CI 1.34-2.12, *p* <0.0001) and Ngerenya late (OR 1.30, 95% CI 0.60-2.81, *p* = 0.5018)". Here is confusing that prior clinical malaria refers to number of malaria episodes, instead of malaria episodes in the previous survey period?

 (Page 13) "We found that before 2006, the number of malaria episodes were associated with an increased risk of gametocyte positivity (OR 1.38, 95% CI 1.15-1.66, *p* = 0.0006) while recent malaria episodes were associated with an approximately threefold increased risk of gametocyte positivity (95% CI 1.85, 4.53, *p* <0.0001)". The second part of the sentence would be more clear: 'while malaria episodes within 28 days of survey were associated….. (OR 2.89, 95% CI 1.85-4.53, p <0.0001)'.

 Table 9 should read 'Number of malaria episodes' instead of 'No of malaria episodes'.

**Discussion:**

 (Page 16, line 3) – 15 years of age (instead of 15 years).

 (Page 16) "Furthermore, it is expected that the prevalence of gametocytaemia would fall in the community as the prevalence of asymptomatic parasitaemia falls ^38^. Moreover, prior episodes of clinical malaria are a well documented source of gametocytaemia ^6,11^. The findings in Junju are therefore unexpected: gametocyte prevalence was disproportionately lower compared with the prevalence of asymptomatic parasitaemia (Figure 4)". Figure 4 shows asexual parasitemia; do the authors mean asymptomatic infections here? This should be clarified, it is now not clear (in the methods and result section) if authors refer to asymptomatic infections or all asexual infections.

 "prior episodes of clinical malaria were modestly protective rather than a risk factor for gametocytaemia. We speculate that a change in anti-malarial drug policy might have caused this variation in effect". Here prior episodes mean episodes within the 28 days of the survey?

 (Page 19) "Interestingly, this and other studies ^16,18^ showed that gametocyte prevalence was highest in 5–15-year-olds in comparison to their younger and older counterparts". The present study does not compare to older than 15 years old.

I have read this submission. I believe that I have an appropriate level of expertise to confirm that it is of an acceptable scientific standard, however I have significant reservations, as outlined above.

# Response

**Published:** 2019-05-19

**Authors:** Michelle Muthui

**Affiliations:** KEMRI-Wellcome Trust Research Programme, Kenya

We would like to sincerely thank you for taking the time to read our manuscript and provide constructive feedback. Please find our responses below:

**Methods/Design:**

- *3 cohorts with weekly follow up of clinical cases (febrile cases) + 1 annual survey for asymptomatic infections.*
*Cross-sectionals surveys – numbers per community and year described in a figure.*
  - *Is the 1 annual survey for asymptomatic infections mentioned in the methods section considered a survey (thus included in Fig 3 and supplementary Table 1)? It was not clear how this data was used.*

Yes, the cross-sectional surveys for asymptomatic infections were conducted within the three cohorts, data from these cross-sectional surveys were then what was used in the analysis to determine parasite prevalence. We have amended this on page 3 in the first paragraph under the sub-heading ‘Study design and data collection’ as below:

‘We analysed data from cross-sectional surveys conducted within the three cohorts. Data included in the analyses were from cross-sectional surveys conducted from 1998 to 2016 for Ngerenya (a cross-sectional survey was not conducted in 2006); from 1999 to 2001 for Chonyi; and from 2007 to 2016 for Junju.’

And the second paragraph as below:

‘Children were actively monitored for malaria by weekly visits to identify febrile episodes and by cross-sectional surveys for asymptomatic parasitaemia…’

- *Through the text and in the tables the authors use different terms such as number of malaria episodes, current and previous survey, prior survey. Some of these terms are confusing. For example:* *Number of malaria episodes occurring between the current survey and the prior survey were then summed up and analyzed as the number of episodes occurring in the current survey."* *Is this variable defined by only weekly follow up data or also cross-sectional surveys?*

The variable ‘number of malaria episodes’ was defined only by the data derived from the weekly surveillance and not data from the cross-sectional parasitological surveys. We have clarified this in the first paragraph under the sub-heading ‘Case definitions’ on page 5 as follows:

‘Malaria episodes occurring in the interval between two cross-sectional surveys, the respective survey (survey x) and the prior survey (survey x-1), were identified, summed up and defined as the number of malaria episodes occurring in the period leading up to each respective survey.’

- *"Previous survey period interval between the preceding survey and the survey that came before."* *Is this using both cross-sectional and weekly follow up data? (Definition is found on page 9 of results).*

The malaria episodes were all calculated from the weekly surveillance and not from the cross-sectional surveys. We agree on reflection that the terminology may not be clear as used and have therefore updated the manuscript as follows:

1. We have amended the first paragraph under the sub-heading ‘Factors predicting gametocyte positivity’ on page 9 to read:‘We tested associations between the following covariates: asexual parasite positivity, age, cohort, number of malaria episodes occurring in the period leading up to a cross-sectional survey and whether an individual was gametocyte positive or asexual parasite positive during the prior survey or had malaria episodes in the period leading up to the prior survey (prior to each respective survey).’
2. We have updated the tables and text as well and replaced ‘Malaria episodes in the previous survey period’ with ‘Number of malaria episodes in the prior survey’. We have also changed ‘Gametocyte positive the previous survey’ with ‘Gametocyte positive in the prior survey’ and ‘Asexual parasite positive the previous survey’ with ‘Asexual parasite positive in the prior survey’. We have also amended the footnote beneath the tables to read: ‘ ^i ^the ‘number of malaria episodes’ is defined as the sum of the number of malaria episodes occurring in the period leading up to a cross-sectional survey’.

- *Asexual parasite positive. Is it all infections or asymptomatic infections only?*

The variable asexual parasite positive refers to all infections that were positive for asexual parasites as detected microscopically during the cross-sectional surveys. We have since amended this on page 5 under the paragraph ‘Statistical analysis’ to read:

‘The variable asexual parasite positive included all infections – asymptomatic and symptomatic.’

- *Malaria episode within 28 days of survey, is this variable the same than recent malaria episodes used in the text*

Yes, the malaria episodes occurring within 28 days of a cross-sectional survey are the same as recent malaria episodes. We realise that the connection between the two terms was missing in the text and have amended the text in the final paragraph of page 13 to read:

‘…while malaria episodes occurring within 28 days of a cross-sectional survey were associated with an approximately threefold increased risk of gametocyte positivity…’

- *Study population: There are two tables presenting characteristics of the study population. Does Table 1 include all participants (both cohorts and cross sectionals) or does it include only cross-sectional? Or are cross-sectional study participants a sub-group of the cohort participants?*

The data in Table 1 represents the characteristics of the study participants who participated in the cross-sectional surveys. Participants were recruited to the three cohorts from which each year over the course of follow-up a cross-sectional survey was conducted and weekly surveillance to monitor for malaria episodes. Therefore, the cross-sectional data is derived from the cohort. We have amended this on page 3 in the first paragraph under the sub-heading ‘Study design and data collection’ as below:

‘We analysed data from cross-sectional surveys conducted within the three cohorts. Data included in the analyses were from cross-sectional surveys conducted from 1998 to 2016 for Ngerenya (a cross-sectional survey was not conducted in 2006); from 1999 to 2001 for Chonyi; and from 2007 to 2016 for Junju.’

- *While in the text the authors state “The demographic characteristics of study participants participating in the cross-sectional surveys are presented in Table 1, the characteristics of the study participants participating in the weekly follow-up visits are provided in Table 2”, Table 1 footer defines malaria episodes as calculated from weekly follow-up data and does not include cross-sectional in the title. This is confusing*

We agree that this may be unclear and have therefore renamed the title of Table 1 to read:

‘Table 1. Demographic characteristics of study participants participating in the cross-sectional surveys’.

The number of malaria episodes were calculated from the weekly follow-up data for the period leading up to each survey and then summed up and the figure assigned to the respective cross-sectional survey. This is what we present in Table 1.

- *Laboratory investigations: Describes an external and internal quality control of slides. Since the study covers 19 years, is this assurance system covering the full period? What was the result?*

Since the start of the cohort follow-up, there have been internal and external quality control systems in place for the microscopy. The nature of quality assurance, however, has changed over time. In the beginning, the internal quality control involved the selection of 10 slides every three months that were then rotated amongst the microscopists. Concordance among the results was checked and for results that deviated from the average range, the responsible microscopist was required to repeat the readings as a training exercise. External quality assurance involved the same microscopists reading reference slides obtained from labs in the United Kingdom (UK) where their results would then be compared to the standard reported for those slides. Over time, however, the internal quality control has changed whereby 10% of the slides read in a quarter are selected at random by an independent member of the lab, the readings repeated by the microscopists and compared for concordance between the new and the old results. For external quality control, this has changed where we now participate in three surveys per year carried out by the National Institute of Communicable Diseases (NICD) based in South Africa where they send 20 slides per survey to our lab for reading. The results are then compared to the reference results provided by NICD.

We have amended the second paragraph on page 5 under the sub-heading ‘Laboratory investigation’ as follows:

‘Quality assurance over the study period included comprehensive microscopy training during induction and at regular intervals using internal and external quality control. For internal quality control, a subset of slides selected quarterly is re-read by the microscopy team and concordance between the results checked. For external quality control, at the beginning of the cohort study, this involved reading reference blood films from a partner lab in the United Kingdom. Currently, external quality control involves participating in three annual surveys carried out by the National Institute of Communicable Diseases (NICD) based in South Africa where they send 20 slides per survey to our lab for proficiency testing.’

**Results:**

- *Page 9) "Prior clinical malaria episodes were associated with increased odds of gametocyte positivity in Chonyi (OR 1.49, 95% CI 1.06-2.12, p = 0.0234), Ngerenya early (OR 1.68, 95% CI 1.34-2.12, p <0.0001) and Ngerenya late (OR 1.30, 95% CI 0.60-2.81, p = 0.5018)". Here is confusing that prior clinical malaria refers to number of malaria episodes, instead of malaria episodes in the previous survey period?*

We accept that the statement could be clarified and have therefore have amended the statement to read on page 9 in the third paragraph to read:

‘The number of malaria episodes occurring in the period leading up to a cross-sectional survey were associated with increased odds of gametocyte positivity in Chonyi …’.

- * (Page 13) "We found that before 2006, the number of malaria episodes were associated with an increased risk of gametocyte positivity (OR 1.38, 95% CI 1.15-1.66, p = 0.0006) while recent malaria episodes were associated with an approximately threefold increased risk of gametocyte positivity (95% CI 1.85, 4.53, p <0.0001)". The second part of the sentence would be more clear: 'while malaria episodes within 28 days of survey were associated….. (OR 2.89, 95% CI 1.85-4.53, p <0.0001)'.*

We agree and have amended the sentence in the last paragraph on page 13 as follows:

‘…while malaria episodes occurring within 28 days of a cross-sectional survey were associated with an approximately threefold increased risk of…’

- *Table 9 should read 'Number of malaria episodes' instead of 'No of malaria episodes'.*

We acknowledge this oversight and have amended Table 9 to read ‘Number..’ instead of ‘No..’.

**Discussion**

- *(Page 16, line 3) – 15 years of age (instead of 15 years).*

We acknowledge this oversight and have edited the text on page 16 line 3 to now read ‘…15 years of age…’.

- *(Page 16) "Furthermore, it is expected that the prevalence of gametocytaemia would fall in the community as the prevalence of asymptomatic parasitaemia falls38. Moreover, prior episodes of clinical malaria are a well-documented source of gametocytaemia6,11. The findings in Junju are therefore unexpected: gametocyte prevalence was disproportionately lower compared with the prevalence of asymptomatic parasitaemia (Figure 4)". Figure 4 shows asexual parasitemia; do the authors mean asymptomatic infections here? This should be clarified, it is now not clear (in the methods and result section) if authors refer to asymptomatic infections or all asexual infections.*

The main purpose of the cross-sectional parasitological surveys is to assess asymptomatic asexual parasite prevalence in the three cohorts as they were carried out at the end of the dry season when parasite transmission is low. For this reason, in the methods section, we refer to asymptomatic infections.

We, however, acknowledge that the terminology may not be accurately described in the discussion and have amended the 2 ^nd^ paragraph of the discussion on page 16 to read:

‘Furthermore, it is expected that the prevalence of gametocytaemia would fall in the community as the prevalence of asymptomatic asexual parasitaemia falls ^38^. Moreover, prior episodes of clinical malaria are a well-documented source of gametocytaemia ^6^^, ^^11^. The findings in Junju are therefore unexpected: gametocyte prevalence was disproportionately lower compared with the prevalence of asexual parasitaemia (Figure 4), and malaria episodes occurring in the period before a cross-sectional survey were modestly protective rather than a risk factor for gametocytaemia (Table 6). We speculate that a change in anti-malarial drug policy might have caused this variation in effect.’

- *"prior episodes of clinical malaria were modestly protective rather than a risk factor for gametocytaemia. We speculate that a change in anti-malarial drug policy might have caused this variation in effect". Here prior episodes mean episodes within the 28 days of the survey?*

Here we refer to the number of malaria episodes occurring in the period leading up to a cross-sectional survey, to improve the clarity of the sentence we have amended it to read:

‘…and malaria episodes occurring in the period before a cross-sectional survey were modestly protective rather than a risk factor for gametocytaemia (Table 6)’.

- * (Page 19) "Interestingly, this and other studies16,18 showed that gametocyte prevalence was highest in 5–15-year-olds in comparison to their younger and older counterparts". The present study does not compare to older than 15 years old.*

In this paragraph, the study we were referring to is the cited study in the previous sentence by Goncalves *et al.* and not the present study. However, we acknowledge that this may not be clear as written and have therefore amended the sentence in the first paragraph of page 19 to read:

 ‘Interestingly, this previous study by Goncalves *et al*. ^48^ and other studies…’
